# How fullerene derivatives (FDs) act on therapeutically important targets associated with diabetic diseases

**DOI:** 10.1016/j.csbj.2022.02.006

**Published:** 2022-02-12

**Authors:** Natalja Fjodorova, Marjana Novič, Katja Venko, Viktor Drgan, Bakhtiyor Rasulev, Melek Türker Saçan, Safiye Sağ Erdem, Gulcin Tugcu, Alla P. Toropova, Andrey A. Toropov

**Affiliations:** a National Institute of Chemistry, Ljubljana, Slovenia; b North Dakota State University, Fargo, ND, USA; c Bogazici University, Institute of Environmental Sciences, Istanbul, Turkey; d Marmara University, Department of Chemistry, Istanbul, Turkey; e Yeditepe University, Faculty of Pharmacy, Istanbul, Turkey; f Laboratory of Environmental Chemistry and Toxicology, Istituto Di Ricerche Farmacologiche Mario Negri IRCCS, Via Mario Negri, 2, Milano 20156, Italy

**Keywords:** Fullerene-based nanoparticles, Fullerene derivatives, Neural network models, Anti-diabetic targets, Protein–ligand binding

## Abstract

Fullerene derivatives (FDs) belong to a relatively new family of nano-sized organic compounds. They are widely applied in materials science, pharmaceutical industry, and (bio) medicine. This research focused on the study of FDs in terms of their potential inhibitory effect on therapeutic targets associated with diabetic disease, as well as analysis of protein–ligand binding in order to identify the key binding characteristics of FDs.

Therapeutic drug compounds when entering the biological system usually inevitably encounter and interact with a vast variety of biomolecules that are responsible for many different functions in organisms. Protein biomolecules are the most important functional components and used in this study as target structures. The structures of proteins [(PDB ID: 1BMQ, 1FM6, 1GPB, 1H5U, 1US0)] belonging to the class of anti-diabetes targets were obtained from the Protein Data Bank (PDB). Protein binding activity data (binding scores) were calculated for the dataset of 169 FDs related to these five proteins. Subsequently, the resulting data were analyzed using various machine learning and cheminformatics methods, including artificial neural network algorithms for variable selection and property prediction.

The Quantitative Structure-Activity Relationship (QSAR) models for prediction of binding scores activity were built up according to five Organization for Economic Co-operation and Development (OECD) principles.

All the data obtained can provide important information for further potential use of FDs with different functional groups as promising medical antidiabetic agents. Binding scores activity can be used for ranking of FDs in terms of their inhibitory activity (pharmacological properties) and potential toxicity.

## Highlights

• Five proteins related to diabetic disease were selected from Protein Data Bank.
• Binding scores were calculated for five proteins with 169 fullerene derivatives.
• Correlation between drug-like descriptors and binding scores activity was examined.
• The contribution of descriptors to protein-ligand binding was demonstrated.
• The QSARs models for prediction of binding scores activity were built.

## Graphical abstract

**Figure:**
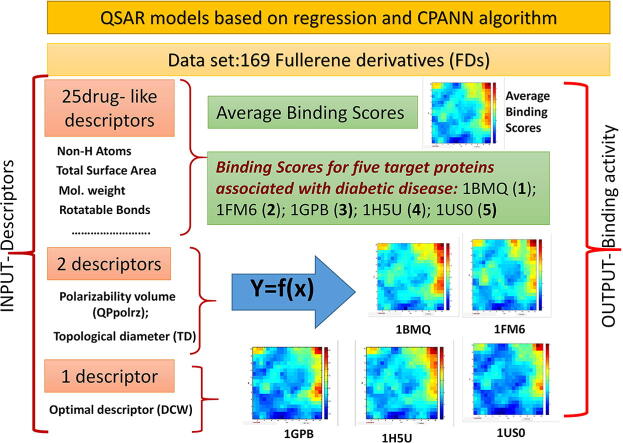

## 1 Introduction

Diabetes is a chronic disease that occurs either when the pancreas does not produce enough insulin or when the body cannot effectively use the insulin it produces. Insulin is a hormone that regulates blood sugar. Hyperglycemia, or elevated blood sugar, is a common consequence of uncontrolled diabetes and over time causes severe damage to many body systems, especially nerves and blood vessels [1]. In 2019, an estimated 1.5 million deaths were directly caused by diabetes. Another 2.2 million deaths were due to high blood glucose levels in 2012. Between 2000 and 2016, there was a 5% increase in premature mortality due to diabetes [1]. The World Health Organization (WHO) estimated that diabetes would be the 7th leading cause of death by 2030 [2]. Diabetes is divided into three main types: Type I, Type II and gestational diabetes. Type II diabetes mellitus (T2DM) accounts for more than 90% of all diabetes cases [3]. T2DM is a heterogeneous disease associated with both genetic and environmental causes, including several defects in insulin secretion and action [4], [5].

Insulin is a hormone that moves glucose into cells to produce energy. When insulin secretion is inadequate, glucose levels in the blood rise (hyperglycemia). Prolonged hyperglycemia causes irreversible damage to the eyes, kidneys, nerves, and heart [6]. A review of antidiabetic drugs and their development has been published in an article [7]. Recent successes in the discovery and development of new targets for the treatment of T2DM were reported in 2021 [8].

The first traditional antidiabetic drugs focused on controlling blood glucose concentration. The next generation of antidiabetic drugs focused on delaying disease progression and treatment failure, which causes poorer glycemic regulation. Recent treatment approaches target several novel pathophysiological defects present in T2DM. Promising new targets in clinical development include those that increase insulin sensitization (glucocorticoid receptor antagonists), decrease hepatic glucose production (glucagon receptor antagonists, glycogen phosphorylase and fructose-1,6-biphosphatase inhibitors). There is limited information on the use of FDs as antidiabetic agents. In the paper by Soldatova et al. [9], it was presented for the first time that the pentaamino acid derivative of fullerene C60 (potassium salt of fullerenylpenta-N-dihydroxytyrosine) affects three targets of T2DM. It competitively inhibits the enzymes aldose reductase and sorbitol dehydrogenase and also has an antiglycation effect on bovine serum albumin. The inhibition constants for these enzymes were demonstrated. The antidiabetic effect of FDs *in vivo* has been described in papers [10], [11]. The authors investigated the efficacy of magnesium-25 carrying porphyrin- fullerene nanoparticles in diabetes-induced neuropathy.

A review on the role of antioxidants in the treatment of diabetes mellitus (DM) and its complications was published in Rahimi et al. [12]. The authors noted that there is growing evidence that increased production and/or ineffective scavenging of reactive oxygen species (ROS) may play a crucial role in certain pathological conditions, especially chronic diseases. The high reactivity of ROS leads to chemical changes in virtually all cellular components, resulting in lipid peroxidation. This review indicates well that oxidative stress is involved in the pathogenesis of DM and its complications. The intake of antioxidants reduces oxidative stress and alleviates diabetic complications.

Fullerene C60 and water-soluble FDs were used as antioxidants against radical-initiated lipid peroxidation which was reported in the study by Wang et al. [13]. FDs can possess antioxidant properties. They have found wide application in medicinal chemistry [14]. Fullerenes are commonly referred to as “radical sponges” [15] due to their remarkable reactivity with free radicals [16], [17], [18], [19]. The radical scavenging properties of FDs have found many applications in biological systems. They are used to treat various biological disorders caused by free radicals. These mainly include neurodegenerative diseases (i.e. amyotrophic lateral sclerosis, Alzheimer's disease, Parkinson's disease) and other cytotoxic processes caused by oxidative stress. The FDs are used as cytoprotective agents against oxidative stress [20]. The FDs can prevent apoptosis by neutralizing reactive oxygen species (ROS).

The ability of FDs to fit inside the hydrophobic cavity of human immunodeficiency virus (HIV) proteases makes them a potentially good inhibitor of the enzyme's catalytic active site. Therefore, FDs have found their application as antiviral drugs [21], [22], [23], [24], [25], [26]. It has been found that the antiviral activity of FDs is due to their antioxidant activity.

Many of the most effective drugs in therapeutic areas such as oncology, psychiatry, inflammation, etc., act on multiple targets rather than just one [27], [28], [29], [30]. The “one drug - one target - one disease” paradigm in drug discovery has been reconsidered in the last decade. This paradigm shift was mainly caused by the high attrition rates in drug approvals due to toxicity and lack of efficacy. Computational techniques play an important role in the prediction and discovery of new targets for approved drugs. In this context, machine learning approaches such as self-organizing maps and inverse distance weighting are used for polypharmacological profiling of bioactive compounds, as shown in the following prospective studies [31], [32], [33], [34].

The study of FDs in the context of anti-diabetes targets may offer a new opportunity to cure this disease. The potential possibility of using FDs as antidiabetic agents was the focus of our research. We investigated the binding score activities of 169 FDs in relation to five anti-diabetes targets: 1BMQ, 1FM6, 1GPB, 1H5U, and 1US0. The average binding scores activity of 169 FDs in relation to 1117 proteins was taken from previous studies [35], [36]. In this study the binding score activity of 169 FDs in relation to five diabetes-related proteins was compared to the average binding score activity of 169 FDs in relation to 1117 proteins. The study showed how FDs affect the individual binding score activity of five diabetes-related proteins, considering the effects of FDs on the overall biological system of 1117 proteins.

We then investigated the key binding characteristics of the fullerene nanoparticles studied in terms of their contribution to the protein–ligand binding. In particular, the contribution of the drug-like descriptors to the binding activity was considered in this article.

The models for the prediction of binding scores activity were developed in accordance with five OECD principles and analyzed.

## 2 Materials and methods

### 2.1 Dataset

In the current study, a dataset of 169 FDs obtained from the literature [35] was examined. The substituent groups are attached to the fullerene core C60. The exceptions are FD50- C70 and FD169- C80H2. FD168 represents pristine C60 without substituent groups.

169 FDs were divided into an active training set (≈25%), a passive training set (≈25%), a calibration set (≈25%), and a validation set (≈25%) using CORAL software (http://www.insilico.eu/coral). In the case of Counter Propagation Artificial Neural Network (CPANN), the active training set, passive training set, and calibration set were combined in the training set. Thus, the training set consisted of 127 compounds, while the test set consisted of 42 compounds.

The structures of proteins [(PDB ID: 1BMQ, 1FM6, 1GPB, 1H5U, 1US0)] belonging to antidiabetic targets were taken from RCSB Protein Data Bank [37]. The properties of these five proteins are listed in Table 1.

**Table 1:** A brief description of five antidiabetic target proteins.

PDB_ID	Target details/function	Organism	Biochemical function/Classification	Related diseases
1BMQ	Interleukin-1 β converting enzyme (ICE)- a novel cysteine protease responsible for the cleavage of pre-interleukin-1β (pre-IL-1β) to the mature cytokine.	Homo sapiens	Enzyme/Hydrolase	Brain inflammation; Cerebral ischemia; Diabetic retinopathy; Inflammation
1FM6	Peroxisome Proliferator Activated Receptor γ (PPAR γ)/The nuclear receptor PPARgamma/RXRalpha heterodimer regulates glucose and lipid homeostasis and is the target for the antidiabetic drugs.	Homo sapiens	Receptor/Transcription	Adrenocorticotrophic Hormonesecreting Pituitary Tumors, Atherosclerosis, Atopic Dermatitis, Autoimmune Diseases, Bladder Cancer, Chronic Inflammatory Diseases, Crohn's Disease, Unspecified, Diabetes Mellitus, Inflammation, Inflammatory Bowel Disease, Insulin Resistance, Ischemic Heart Disease, Obesity, Pancreatic Cancer, Psoriasis…
1GPB	Glycogen Phosphorylase B, Muscle Form- one of the phosphorylase enzymes. Glycogen phosphorylase catalyzes the rate-limiting step in glycogenolysis in animals by releasing glucose-1-phosphate from the terminal alpha-1,4-glycosidic bond.	Oryctolagus cuniculus	Enzyme/Glycogen phosphorylase	Diabetes Mellitus; Noninsulindependent Diabetes Mellitus
1H5U	Glycogen phosphorylase B complexed with glucose and cp320626- a potential antidiabetic drug	Oryctolagus cuniculus	Enzyme/Glycogen metabolism	Diabetes mellitus
1US0	Aldose Reductase-a potential antidiabetic drug, inhibits glycogen phosphorylase in synergism with glucose	Homo sapiens	Enzyme/ Oxidoreductase	Diabetic neuropathy; Diabetic retinopathy; Neuropathic pain; Noninsulindependent diabetes mellitus; Diabetic complications.

In order to develop models a set of several types of descriptors was generated and applied.

First, two important descriptors with physical meaning obtained from the study of Ahmed et al. [36] were applied in the study. The first descriptor is polarizability given as polarizability volume in cubic angstroms (QPpolrz)) and the second descriptor is topological diameter (TD), characterized the size of the molecules and correlated with the binding activity.

Second, the Monte Carlo descriptors or so-called optimal descriptors (DCW) [38] were generated using the software CORAL (http://www.insilico.eu/coral). These descriptors are the basis for Monte Carlo models suitable for modelling various endpoints [39], [40], in particular for FDs [41], [42]. In the Monte Carlo method the Simplified Molecular Input-Line Entry System (SMILES) is used as representation of molecular structure. The conversion of SMILES into molecular graph for Quantitative Structure-Activity Relationship (QSAR) analysis was performed using CORAL software. Optimal descriptors can be a translator of eclectic information into endpoint prediction [39], [40].

Third, the pharmaceutically relevant properties of FDs were calculated using DataWarrior software (Actelion Pharmaceuticals Ltd., Allschwil, Switzerland) [43]. The following twenty-five descriptors were used for modelling: H-acceptors, H-donors, total surface area, relative polar surface area (RPSA), polar surface area (PSA), drug-likeness, molecular weight, cLogP, cLogS, electronegative atoms, stereo-centers, rotatable bonds, ring closures, small rings, aromatic rings, aromatic atoms, sp3- atoms, symmetric atoms, amides, amines, aromatic nitrogen, basic nitrogen, acidic oxygens, non-H atoms, non-C/H atoms.

The analysis of drug-like properties of FDs was carried out in our study to determine the relationship between them and binding activity as well. The concept of drug-likeness provides useful guidelines for early-stage drug discovery [44], [45]. It involves the analysis of the observed distribution of some key physicochemical properties of approved drugs, including molecular weight, hydrophobicity and polarity, which are related to known drugs [46].

Calculated descriptors used in this study are explicable to researchers involved in drug design, and for the future study of FDs that are promising for application in drug design.

The assessment of drug-likeness is known as Lipinski's Rule of Five (Ro5), which uses simple counting criteria (such as thresholds for molecular weight, log P, or the number of hydrogen bond donors or acceptors) and others [47]. The “drug-like” properties include structural features and physicochemical properties. These properties can be used to characterize the pharmacophore: a substituent in FDs or a part of a molecular structure responsible for a particular biological or pharmacological interaction [48]. The presence of various pharmacophore features affects the behavior of the molecule in a living organism, including bioavailability, transport properties, affinity for proteins, reactivity, toxicity, metabolic stability, and many others.

### 2.2 The Counter Propagation Artificial Neural network algorithm and self-organizing Kohonen maps

**Fig. 1:**
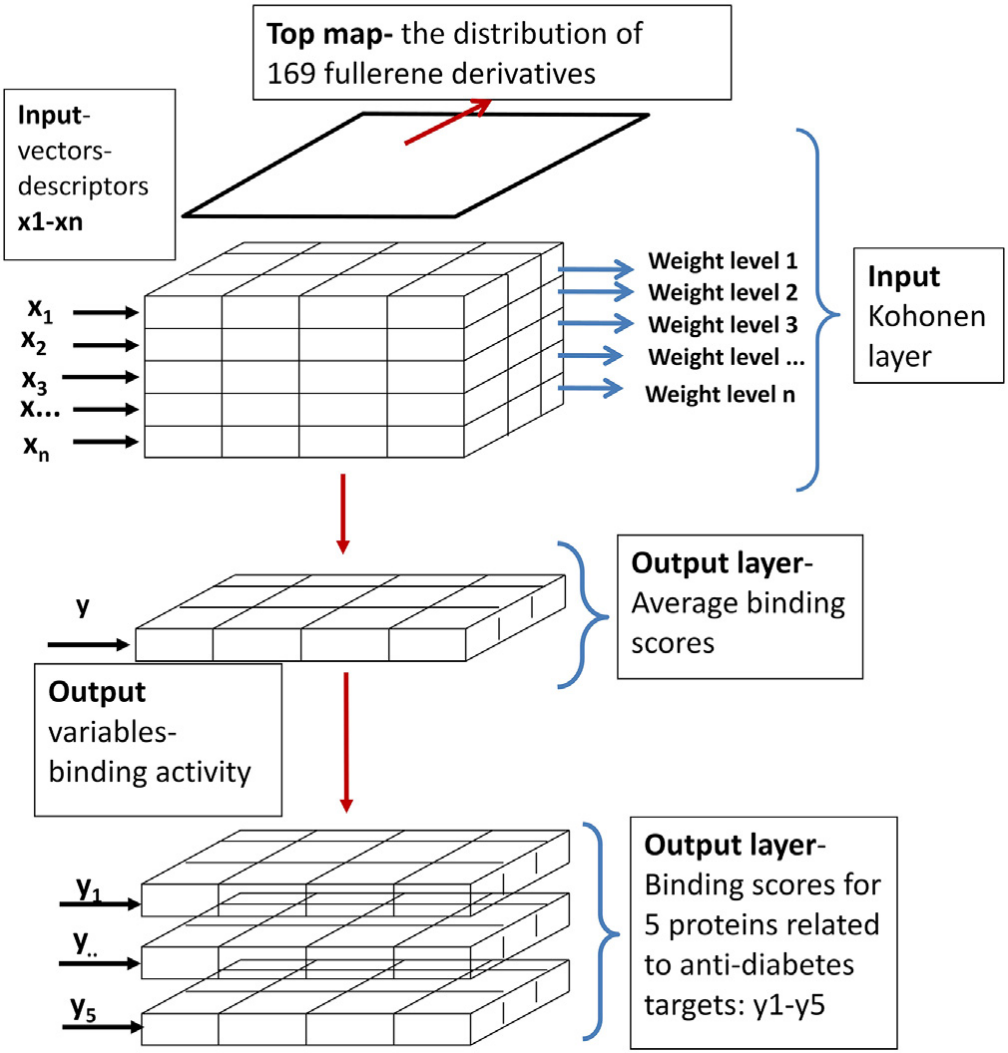
The architecture of CPANN.

The architecture of Counter Propagation Artificial Neural Network (CPANN) used in this study is shown schematically in Fig. 1.

CPANNs are one of the self-organizing mapping techniques commonly used to analyse multidimensional data. The basis of this technique is a nonlinear projection from multidimensional space onto a two-dimensional map. The topology-preserving projection is achieved during training by a nonlinear algorithm. During the training of the network, similar objects are placed close to each other. Therefore, it is expected that chemicals with similar structures or similar properties form clusters on the two-dimensional map [49].

The architecture of CPANN shown in Fig. 1 consists of two layers: the input layer (Kohonen layer) of neurons contains encoded information from molecular structures described with molecular descriptor values, and the output layer is related to binding score activity. Both layers of neurons are exactly superimposed and the output layer has exactly the same arrangement of neurons as the input layer [49], [50], [51], [52]. In Fig. 1, the inputs x_1_- x_n_ are vector components corresponding to *n* descriptors computed for all FDs in the set used for training. The training was performed using the in-house developed TRACEANN toolbox for Matlab [53], which is available online (https://www.ki.si/en/departments/d01-theory-department/laboratory-for-cheminformatics/software/). The toolbox performs classification of multivariate data using the Kohonen mapping method and predictive modelling using CPANN, which includes visualization (contour plots, 3D visualization, and coloured neurons) of the Kohonen levels. The self-organizing Kohonen maps are used as a data visualization technique [54] to visualize structurally similar molecules that tend to have similar activities.

### 2.3 Regression analysis

Regression analysis was used to estimate the relationships between a dependent variable (response = binding score activity) and independent variables (descriptors including drug-like descriptors). Statistical models explain the biological activity of ligands (FDs). Regression analysis was performed using Minitab statistical software. The plots of actual vs. predicted binding score activity were obtained.

### 2.4 Domain of applicability

In order to verify the applicability domain (AD) of our QSAR models, we applied the leverage approach [55]. Leverages are measures of the distance between the x-values for one observation and the mean of the x-values for all observations. In terms of the variables used in our study, this approach provides a measure of the distance between the descriptor values for one chemical and the mean of the descriptor values for all chemicals. A large leverage value indicates that the x-values for one observation are far from the center of the *x*-values for all observations. The leverage *h* of a compound measures its influence on the model. The warning leverage (h*) is generally set to 3(*p* + 1)/*n*, where *n* is the number of training chemicals and *p* is the number of model variables (descriptors) plus one.

## 3 Results and discussions

### 3.1 The characteristics of binding activity

Binding activity was expressed as a binding score (Bscore). This variable accounts for several types of intermolecular interactions and evaluates the strength of interaction between protein and ligand (FD). Binding scores were obtained and described in detail in the study of Ahmed et al. [36], where used protein–ligand docking. Proteins were prepared for docking followed by ligand removal from the original (downloaded from PDB) structures. Two types of docking approaches: PatchDock [56] and AutoDock Vina [57] were utilized. The docking [36] was performed by inverse docking computation. Within a set of ligands for a set of targets, inverse docking is a very useful approach to find putative ligands for a specific protein. In this context, the PatchDock was applied for inverse docking strategy. All initial docking models were obtained by employing PatchDock which is based on the local shape feature matching with less steric clashes. Another docking tool, AutoDock Vina, [57] was employed in the study [36] to analyze final docked models and evaluate the H-bond interactions in the binding sites.

In the current study, first, the average value of binding scores for 1117 proteins (referred to as Average Bscores) calculated for each of the 169 FDs was taken from previous work [35]. Second, the binding scores for five anti-diabetes protein targets (1BMQ, 1FM6, 1GPB, 1H5U, 1US0) were calculated using methods described in the article [36].

**Fig. 2:**
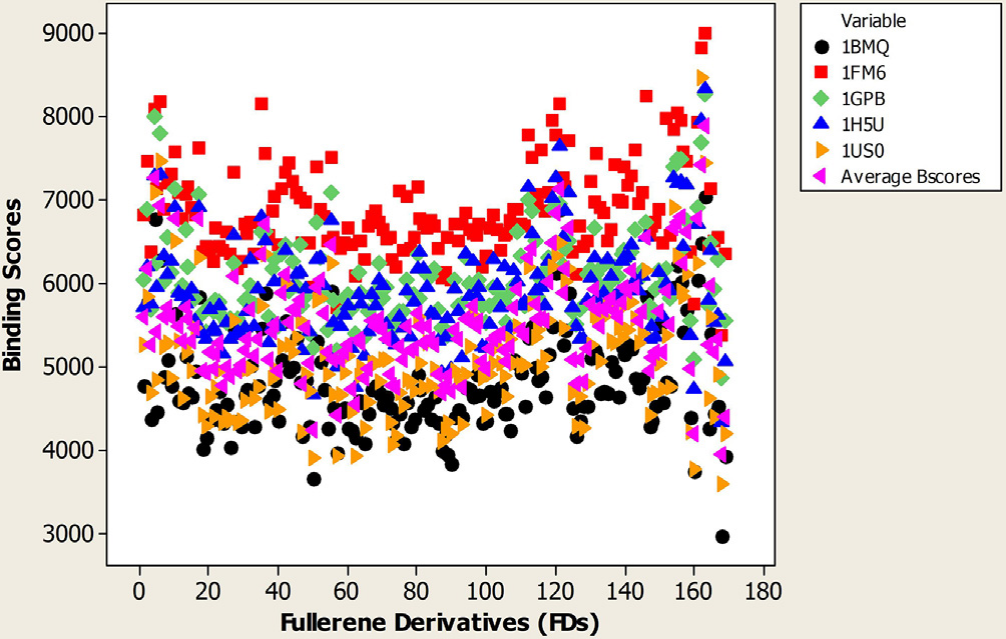
Average binding scores and binding scores related to proteins relevant to diabetic disease (1US0, 1H5U, 1GPB, 1FM6, 1BMQ) for 169 FDs.

Fig. 2 shows the average binding scores and the binding scores with respect to proteins relevant to diabetic diseases (1US0, 1H5U, 1GPB, 1FM6, 1BMQ) for 169 FDs.

It should be emphasized that authors [58] compared the experimental binding affinities (binding energy (BE)) for 20 FDs with calculated one using docking calculations on AutoDock Vina [57] and Schrodinger Suite (Glide sub-program) [59]. They demonstrated a high correlation between the calculated and experimental data (best predictive ability (R^2^training = 0.882 and R^2^test = 0.738)). The whole dataset used for external validation was composed of 49 FDs. The experimental data were correlated with calculated one using docking calculations.

The binding score activity in the present study can be used to rank FDs by their binding score activity in relation to proteins relevant to diabetic disease.

### 3.2 The CPANN model for evaluating the relationships between average binding scores activity, binding scores activity for five proteins associated with diabetes and descriptor values

The CPANN consists of a Kohonen layer (influenced by the input (descriptors)) and an output layer (influenced by the target (binding activity—Binding Scores)). Table 2 shows the input and output variables considered in the study.

**Table 2:** Input variables (containing descriptors) and output variables (containing binding scores activities) of CPANN models.

Input variables	Two descriptors: polarizability volume in cubic angstroms (QPpolrz) (1) and topological diameter (TD) characterized the size of molecules (2);
	Monte Carlo descriptors or so-called optimal descriptor (DCW) (1);
	Twenty five drug like descriptors: H-acceptors (1), H-donors (2), total surface area (3), relative PSA (4), polar surface area (5), drug-likeness (6), Mol. Weight (7), cLogP (8), cLogS (9), electronegative atoms (10), stereo centers (11), rotatable bonds (12), rings closures (13), small rings (14), aromatic rings (15), aromatic atoms (16), sp3-atoms (17), symmetric atoms (18), amides (19), amines (20), aromatic nitrogen (21), basic nitrogen (22), acidic oxygens (23), non-H atoms (24), non-C/H atoms (25).

Output variables	Average Binding Scores (1)
	Binding Scores for five proteins related to diabetic disease:1BMQ (1)- Enzyme/Hydrolase;1FM6 (2)- Receptor/Transcription;1GPB (3)- Enzyme/Glycogen phosphorylase;1H5U (4)- Enzyme/Glycogen metabolism;1US0 (5)- Enzyme/ Oxidoreductase.

In the first part of the study, we applied the optimal CPANN model with 20x20 neurons trained for 600 epochs. After training CPANN, we obtained a self-organizing Kohonen map in which the position of objects was organized in such a way that the nearest neighbors in the plane were the most similar objects in the dataset. We considered the distribution of FDs on the top-map, distribution of descriptors values in weight maps for each descriptor, and distribution of values of binding scores activities (responses) for each of the output variables. Therefore, the CPANN model was used as a lookup table in this part of the study.

**Fig. 3:**
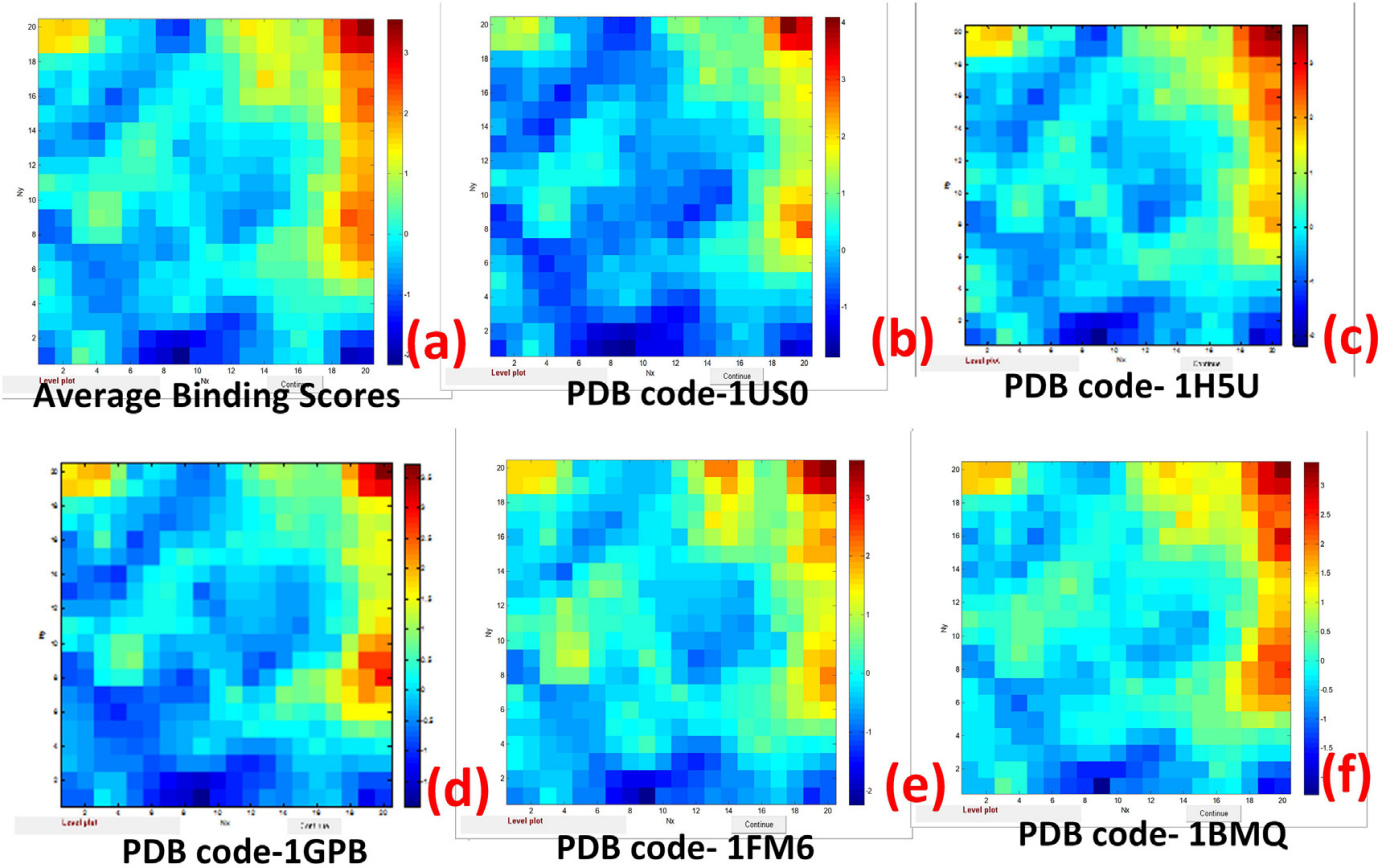
Weight maps for average binding scores **(a)** and binding scores for five proteins relevant to diabetes: 1US0 **(b)**, 1H5U **(c)**, 1GPB **(d)**, 1FM6 **(e)**, and 1BMQ **(f)**.

In this part, we focused on relationships (correlations) and/or similarities between output variables related to binding score activity. As an output (target), the following binding score activity characteristics were considered: Average Binding Scores (1), Binding Scores for five proteins relevant to diabetes: 1BMQ (2); 1FM6 (3); 1GPB (4); 1H5U (5); and 1US0 (6). The statistical performance of the CPANN model is shown in Table S1 in the Supplementary Materials. The squared correlation coefficients R^2^ for the output variables were obtained in the range from 0.988 and 0.957, and the root-mean-squared error (RMSE) ranged from 0.110 and 0.206. Weight maps for average binding scores for all proteins in the dataset and binding scores for five proteins relevant to diabetes (1BMQ, 1FM6, 1GPB, 1H5U, and 1US0) are shown in Fig. 3.

The dark red color corresponds to the highest values of binding scores, while the dark blue color corresponds to the lowest values. The similarity of the color distribution between the weight maps in Fig. 3 shows a high correlation between all the selected variables in terms of binding score activity.

The high correlation between average binding scores and binding scores for five proteins relevant to diabetes [(PDB ID: 1BMQ, 1FM6, 1GPB, 1H5U, 1US0)] was confirmed by calculating Pearson correlation coefficients, which ranged from 0.921 to 0.958.

**Fig. 4:**
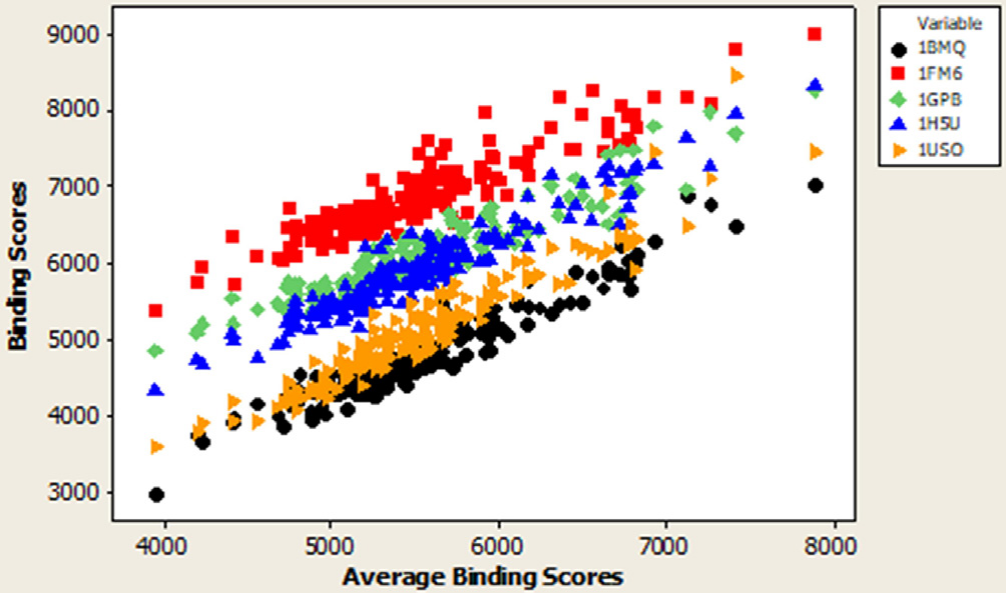
The Binding Scores activity of five proteins associated with diabetes (1BMQ, 1FM6, 1GPB, 1H5U, 1US0) vs. Average Binding Scores activity.

The relationships between binding scores activity of five proteins associated with diabetes (1BMQ, 1FM6, 1GPB, 1H5U, and 1US0) vs. average binding scores activity is illustrated in Fig. 4. The graph shows the correlation between the binding score activity of the proteins associated with diabetic disease and average binding scores activity.

In the second part of the study, we focused on the relationships (correlations) between the descriptors (input variables in the CPANN model) and binding score activities (output (target) variables of the model) listed in Table 2. In other words, it was shown the influence of the most significant descriptors on protein–ligand binding activity which contributes to a mechanistic interpretation of our models.

The Pearson correlation coefficients between all considered descriptors and binding scores activities were calculated using Minitab statistical program. The summary results for the most correlated descriptors (input variables) and binding score activities (output variables) were transformed into summary correlation matrix shown in Table 3. The appropriate range of correlation coefficients related to relationships between binding score activity of five related to diabetic proteins, average binding scores and descriptors is shown in this table.

**Table 3:** Summary matrix with the ranges of correlation coefficients which describe relationships between binding score activities of five diabetic related proteins, average binding scores and descriptors.

Descriptors and responses	Binding scores for five proteins related to diabetic disease [(PDB ID: 1BMQ, 1FM6, 1GPB, 1H5U, 1US0)]
Average Binding Scores	0.921–0.958
Binding scores for five proteins related to diabetic disease [(PDB ID: 1BMQ, 1FM6, 1GPB, 1H5U, 1US0)]	0.880–0.918
Non H-atoms	0.700–0.798
Rotatable Bonds	0.684–0.747
Molecular weight	0.657–0.767
Total Surface Area	0.763–0.859
Topological Diameter (TD)	0.851–0.883
QPpolrz	0.863–0.906
DCW	0.858–0.920

**Fig. 5:**
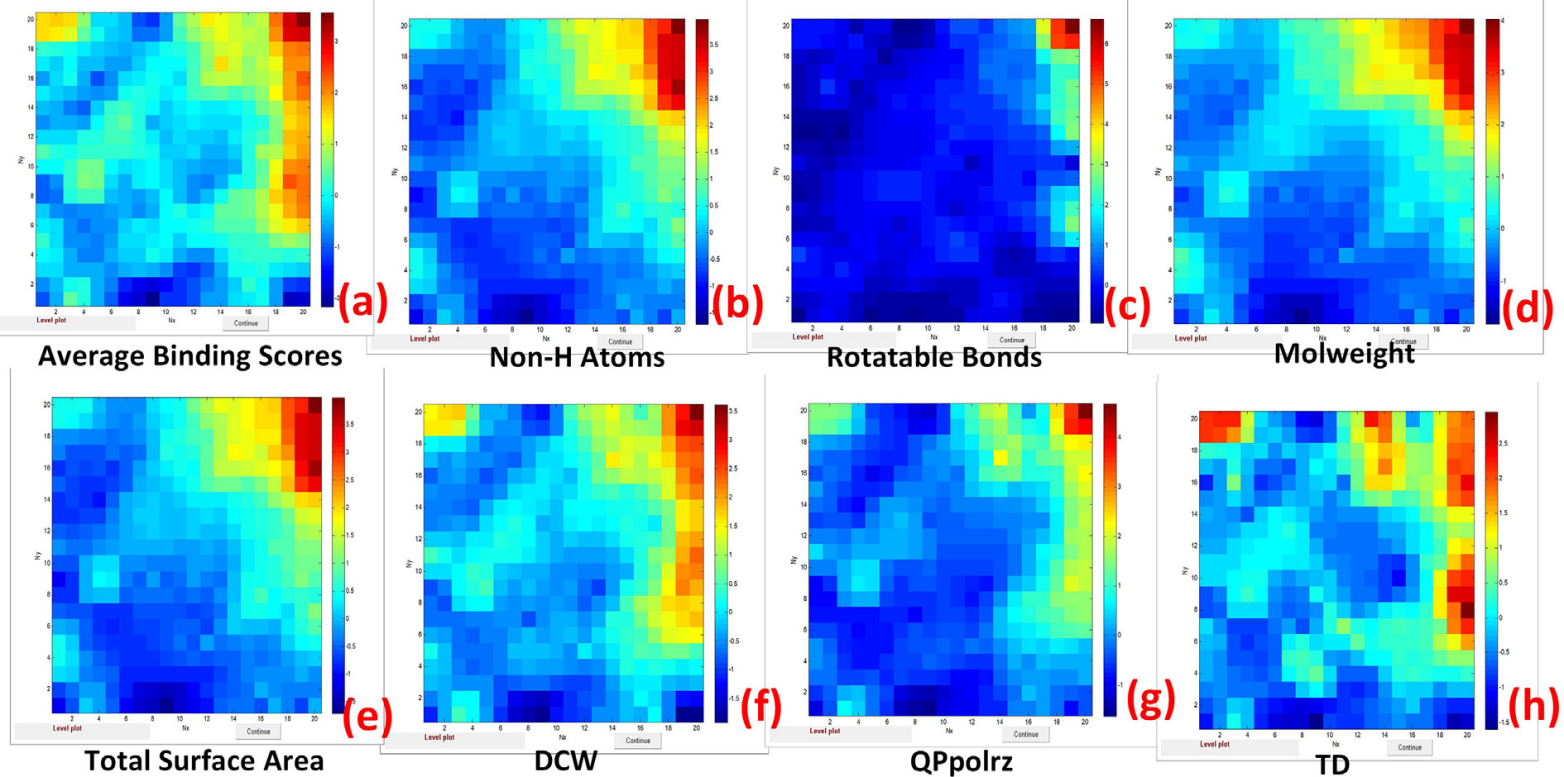
Weight maps of average binding scores **(a)** and the following descriptors: Non H-Atoms **(b)**, Rotatable Bonds **(c)**, Molecular weight **(d)**, Total Surface Area **(e)**, optimal descriptors (DCW) **(f)**, polarizability volume in cubic angstroms (QPpolrz) **(g)**, and topological diameter (TD) **(h)**.

The correlation coefficients between the binding score activity of five proteins associated with diabetes and average binding scores were in the range of 0.921–0.958, as shown in Table 3. The correlation between the binding score activity of five proteins (1BMQ, 1FM6, 1GPB, 1H5U, and 1US0) appeared to be in the range of 0.880–0.918. The correlation between the binding score activity of five proteins associated with diabetes and the descriptors (Non H-atoms, Rotatable Bonds, Molecular Weight, Total Surface Area, Topological Diameter, QPpolrz and DCW) was in the range of 0.657–0.920. This high correlation was illustrated using weight maps for average binding scores and the following descriptors: Non H-Atoms, Rotatable Bonds, Molecular weight, Total Surface Area, optimal descriptor (DCW), polarizability volume in cubic Angstroms (QPpolrz) and topological diameter (TD) (see Fig. 5). The similarity of the weight maps confirms the high correlation between the variables.

Concerning the mechanistic interpretation of the obtained model, we can conclude that the most significant contribution to the protein–ligand binding belongs to the following descriptors: Non H-Atoms, Rotatable Bonds, Molecular weight, Total Surface Area, optimal descriptor (DCW), polarizability volume in cubic Angstroms (QPpolrz) and topological diameter (TD).

In the Supplementary Materials section in Figure S3 we illustrated the distribution of FDs in the top map 20x20 of the CPANN model overlapped with the output layer with binding activity with an indication of the most and least active FDs.

Figure S4 demonstrated the contribution of descriptors: Basic Nitrogens, sp^3^ atoms, Amines, Non H-atoms, Rotatable bonds, Molweight, Total Surface Area, QPpolrz, DCW to the active group of FDs (GROUP A) connected to C60 core with cyclopropane ring containing ammonium groups NH_3_^+^ . The weight maps of mentioned above descriptors show the highest values of these descriptors in this area related to this GROUP A.

Figure S5 demonstrated the contribution of descriptors: Non-C/H Atoms, Acidic oxygens, Electronegative Atoms, Polar Surface Area, H-Acceptors, and Topological Diameter to the active group of FDs (GROUP B) connected to C60 core with a benzene ring and containing nitrogroups-NO_2_. The weight maps of mentioned above descriptors show the highest values of these descriptors in this area related to this GROUP B.

Figure S6 demonstrated the contribution of descriptors: cLogP, Topological Diameter to the active group of FDs (GROUP C) attached to the C60 core with cyclopropane 3-membered ring and containing two benzene rings. The weight maps of mentioned above descriptors show the highest values of these descriptors in this area related to this GROUP C. These two benzene rings are related to endocrine disruptor structural alert.

Figure S7 demonstrated the contribution of descriptors: Basic Nitrogens, Aromatic Nitrogens, Topological Diameter to the active group of FDs (GROUP D) connected to C60 core with pyrrolidine 5-membered ring and containing nitroaromatic substituent. The weight maps of mentioned above descriptors show the highest values of these descriptors in this area related to this GROUP D.

### 3.3 Essential descriptors affecting the binding of FDs to diabetes-associated proteins

Regression analysis was performed to determine essential descriptors affecting the binding scores activity of FDs related to five diabetic disease proteins as well as affecting the average binding scores activity. What descriptor’s characteristics are the most significant in protein–ligand binding?

The summary of the regression analysis using all descriptors, including drug-like descriptors, can be found in Table S2 in the Supplementary Materials. Table S2 contains the regression equations for the responses: average binding scores and binding scores for the five proteins (1BMQ, 1FM6, 1GPB, 1H5U, and 1US0).

The significant contribution to the average binding score activity belongs to the following descriptors: DCW, QPpolrz, topological diameter (TD), H-Acceptors, Total Surface Area, Relative PSA, Molweight, cLogP, Electronegative Atoms, Stereo Centers, Rings Closures, Small Rings, Aromatic Rings, Aromatic Atoms, sp^3^-Atoms, and Non-H Atoms.

In the case of protein 1BMQ (Enzyme/Hydrolase), QPpolrz, topological diameter (TD), Aromatic Nitrogens, and Acidic Oxygens contribute significantly to the binding activity.

The largest contribution in the case of protein 1FM6 (Receptor/Transcription) belongs to the descriptors: QPpolrz, topological diameter (TD), cLogP, Aromatic Rings, Aromatic Atoms, and Aromatic Nitrogens.

For protein 1GPB (Enzyme/Glycogen phosphorylase), the largest contribution to binding activity belongs to the following descriptors: QPpolrz, topological diameter (TD), H-Acceptors, Relative PSA, Molweight, cLogP, cLogS, Electronegative Atoms, Rotatable Bonds, sp3-Atoms, and Non-H Atoms.

In the case of protein 1H5U (Enzyme/metabolism), the main contribution belongs to the following descriptors: topological diameter (TD), H-Acceptors, Electronegative Atoms, Stereo Centers, and sp3-Atoms.

While, in the case of protein 1US0 (Enzyme/oxidoreductase), the major contribution to binding activity belongs to the following descriptors: topological diameter (TD), H-acceptors, H-donors, relative PSA, polar surface area, molecular weight, stereo centers, rotatable bonds, aromatic atoms, sp^3^-Atoms, acidic oxygen atoms, and Non-H atoms.

The largest contributor in all cases is the topological diameter (TD). The size of FDs is significant for all responses: average Bscores and binding scores for five proteins associated with diabetes. This is followed by QPpolrz, which was excluded in the case of the 1H5U and 1US0 proteins.

The coefficients of determination R^2^ in the regressions considered were 0.968 in the case of average binding scores and from 0.857 to 0.895 for the binding scores of five proteins: 1BMQ, 1FM6, 1GPB, 1H5U, and 1US0.

Next, we built regression models for the prediction of binding scores activity based on the two descriptors QPpolrz and TD (Model 1) and the optimal Monte Carlo descriptor DCW (Model 2).

### 3.4 Regression models for predicting binding scores using descriptors QPpolrz and TD (Model 1a) and the optimal descriptors DCW (Model 2a)

The following prediction models were built in the study:

(1) Model 1a for predicting binding activities based on descriptors QPpolrz and TD;

(2) Model 2a is based on the optimal Monte Carlo descriptors DCW generated by the program CORAL.

The regression equations and statistical performance of Model 1a are presented in Table S3 in the Supplementary Materials. The coefficient of determination R^2^ was 0.93 for average binding scores and in the range of 0.81–0.87 for the binding scores activity of the five proteins associated with diabetic disease. The regression analysis (regression equations) and statistical performance of Model 2a are presented in Table S4 in the Supplementary Materials section. The coefficient of determination R^2^ was 0.93 in the case of average binding scores and ranged from 0.74 to 0.85 for the binding activity of five proteins associated with diabetic disease. The plots of actual response vs. predicted were generated for Model 1a (see Fig. 1S) in the Supplementary Materials section and for Model 2a (see Fig. 2S). The plots of actual response vs. predicted show how well our model fits and predicts each observation. The points in all plots show a linear pattern, indicating that the model fits the data well and predicts the response accurately.

In the next part of the study, we decided to build CPANN models for predicting the binding scores activities. The prediction capabilities of the two algorithms were compared to select the best one.

### 3.5 CPANN models for prediction of binding scores activity using the descriptors QPpolrz and TD (Model 1b) and the optimal descriptors DCW (Model 2b)

The CPANN algorithm was used to develop high-quality predictive QSAR models for predicting the binding activity of FDs using two molecular descriptors QPpolrz and Topological Diameter (TD) (Model 1b = M1b) and optimal Monte Carlo descriptors (Model 2b = M2b). The input data for 169 FDs were normalized. The training set consisted of 127 FDs, while the test set consisted of 42 FDs. Internal validation of the CPANN models was performed using the LOO-CV procedure to evaluate the quality and goodness of fit of the model [55], [60].

The optimal CPANN Model 1b with 14x14 neurons was built using two descriptors, QPpolrz and TD, and trained for 400 learning epochs. The optimal CPANN Model 2b was also built with 14x14 neurons using optimal descriptors DCW and trained for 500 learning epochs. The performances of the models are shown in Table 4, Table 5, Table 6 for Average Binding Scores (1) and binding scores for 1BMQ (2), 1FM6 (3), 1GPB (4), 1H5U (5), and 1US0 (6). The best CPANN model built using QPpolrz and TD descriptors (Model 1b) is characterized by a squared regression coefficient for the training set (n = 127) R^2^ = 0.98392 (RMSE = 0.12637), for the test set (n = 42) Q^2^ = 0.99960 (RMSE = 0.01944), leave-one-out cross-validation (LOO-CV) regression coefficient Q^2^cv = 0.97814 (RMSE = 0.14745) related to the average binding scores.

**Table 4:** The statistical performance of models M1b and M2b related to training set.

Output variables	R^2^_M1b_ Training	RMSE_M1b_ Training	R^2^_M2b_ Training	RMSE_M2b_ Training
Average Binding Scores (1)	0.98392	0.12637	0.97968	0.14205
Binding Scores for 1BMQ (2)	0.96423	0.18857	0.95680	0.20709
Binding Scores for 1FM6 (3)	0.96914	0.17518	0.93628	0.25164
Binding Scores for 1GPB (4)	0.95089	0.22095	0.92448	0.27386
Binding Scores for 1H5U (5)	0.94699	0.22948	0.94189	0.24019
Binding Scores for 1US0 (6)	0.97063	0.17078	0.96301	0.19164

**Table 5:** The statistical performance of models M1b and M2b related to test set.

Output variables	Q^2^_ M1b_test	RMSE_M1b_test	Q^2^_M2b_test	RMSE_M2b_test
Average Binding Scores (1)	0.99960	0.01944	0.99895	0.00364
Binding Scores for 1BMQ (2)	0.99511	0.07123	0.99130	0.01220
Binding Scores for 1FM6 (3)	0.98730	0.11072	0.99926	0.00225
Binding Scores for 1GPB (4)	0.99375	0.06871	0.99946	0.00193
Binding Scores for 1H5U (5)	0.99439	0.07745	0.99872	0.00389
Binding Scores for 1US0 (6)	0.99151	0.10027	0.99885	0.00520

**Table 6:** The statistical performance of models M1b and M2b related to validation leave one out (LOO) procedure.

Output variables	Correlation coefficient Q^2^cv_M1b_LOO	RMSE_M1b_ LOO	Correlation coefficient Q^2^cv _M2b_LOO	RMSE_M2b_ LOO
Average Binding Scores (1)	0.97814	0.14745	0.97047	0.17134
Binding Scores for 1BMQ (2)	0.95209	0.21829	0.94056	0.24311
Binding Scores for 1FM6 (3)	0.93697	0.25049	0.90163	0.31278
Binding Scores for 1GPB (4)	0.93066	0.26267	0.90951	0.29999
Binding Scores for 1H5U (5)	0.93188	0.26032	0.93508	0.25406
Binding Scores for 1US0 (6)	0.95677	0.20738	0.92162	0.27916

The best CPANN model using the optimal descriptors DCW (Model 2b) was characterized by a squared regression coefficient for the training set (n = 127) R^2^ = 0.97968 (RMSE = 0.14205), for the test set (n = 42) Q^2^ = 0.99895 (RMSE = 0.00364), leave-one-out cross-validation (LOO-CV) regression coefficient Q^2^cv = 0.97047 (RMSEcv = 0.17134) related to the average binding scores.

High statistical performance was also obtained for the binding scores activities of five proteins associated with the diabetic disease. For the results, see Table 4, Table 5, Table 6.

The model with QPpolrz and TD descriptors (M1b) has only slightly higher performance than the model M2b based on the optimal descriptors DCW.

### 3.6 Domain of applicability of QSAR models

**Fig. 6:**
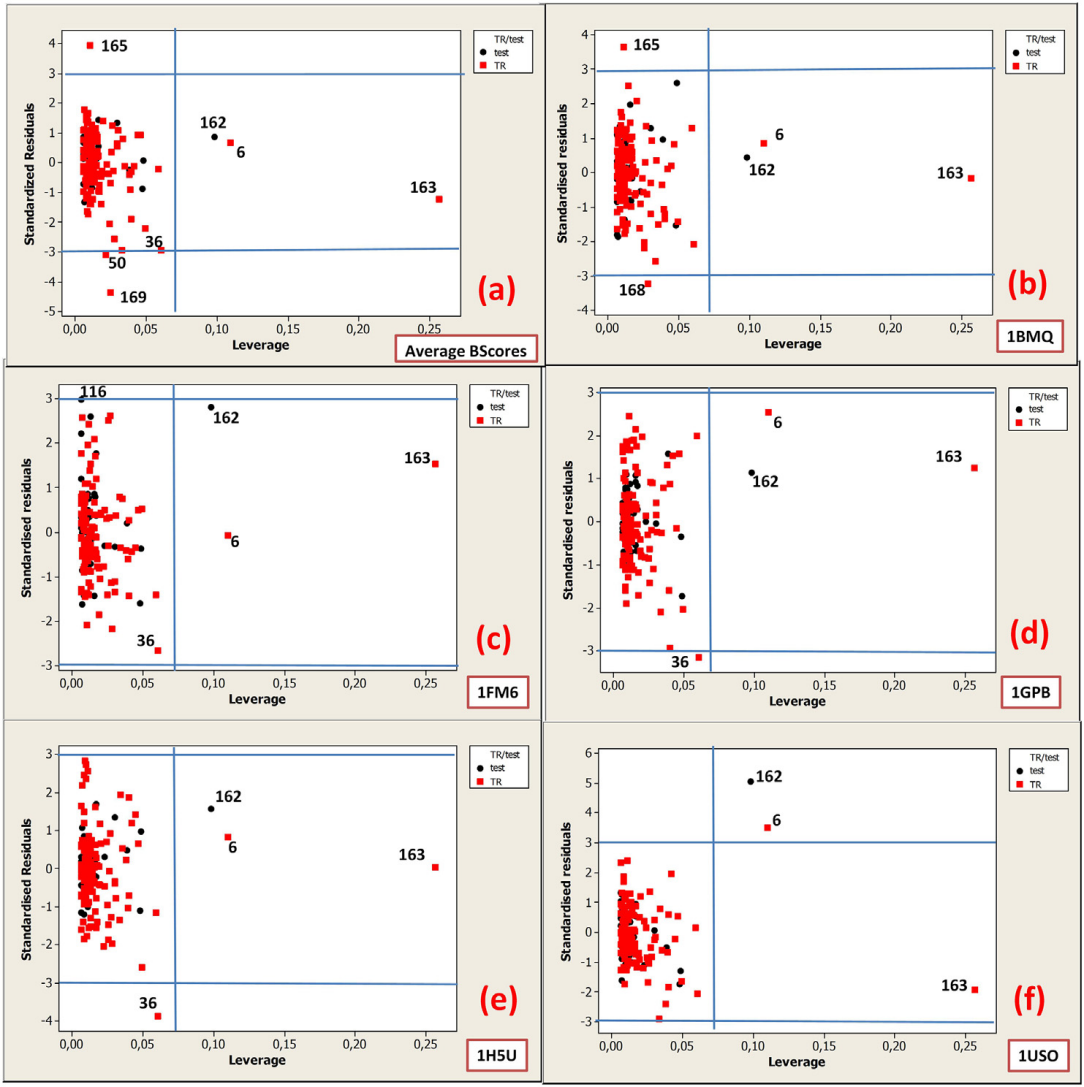
Williams plots: standardized residuals versus leverage for Model 1a based on QPpolrzb and topological diameter (TD) for the following responses: Average BScores **(a)** and binding scores for proteins: 1BMQ **(b)**, 1FM6 **(c)**, 1GPB **(d)**, 1H5U, and 1USO **(f)**.

**Fig. 7:**
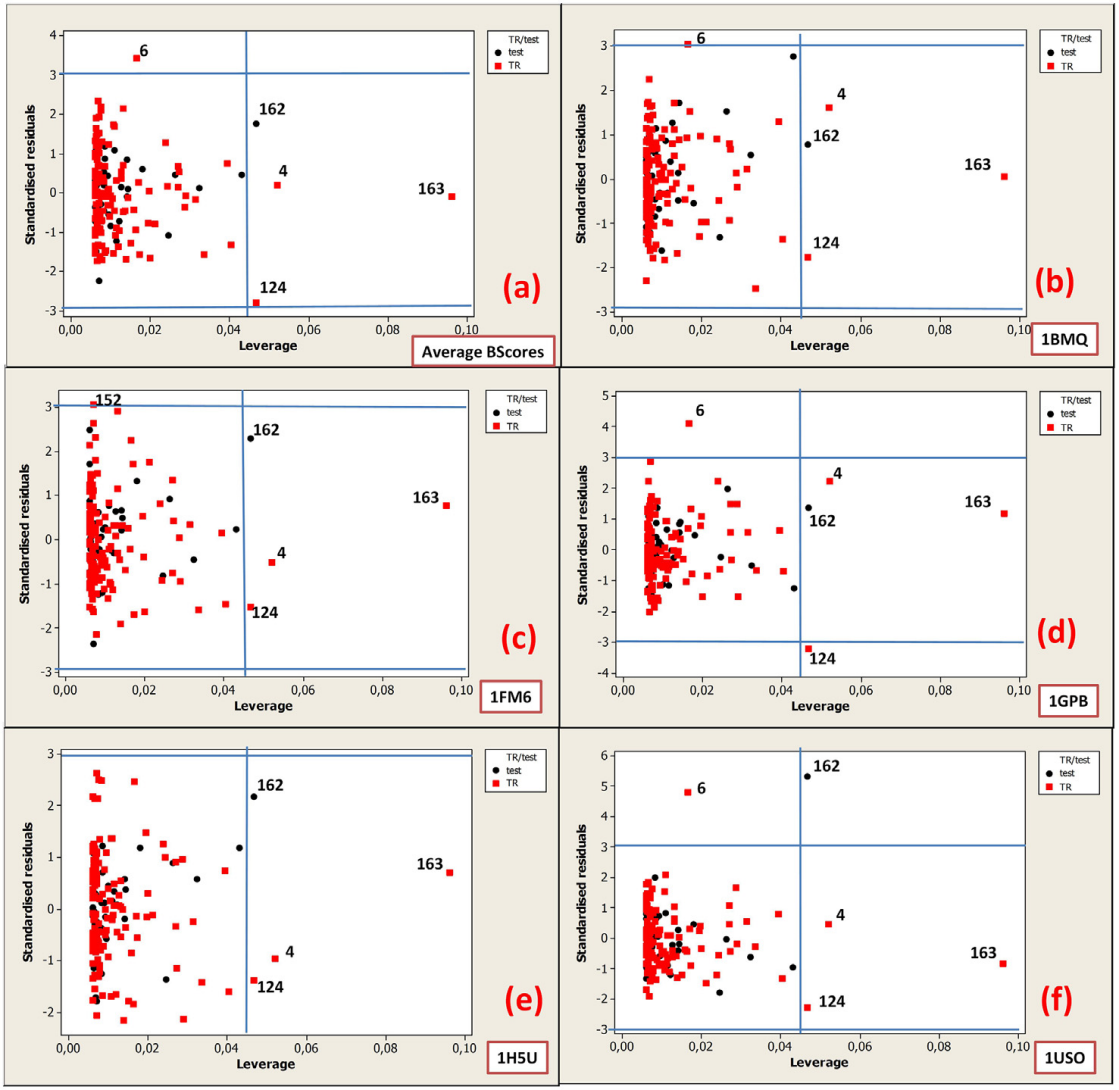
Williams plots: standardized residuals versus leverage for Model 2a based on the optimal Monte Carlo descriptors for the following responses: Average BScores **(a)** and binding scores for proteins: 1BMQ **(b)**, 1FM6 **(c)**, 1GPB **(d)**, 1H5U **(e)**, and 1USO **(f)**.

To visualize the applicability domain (AD) of QSAR models, Williams plots were used where the leverage values (or hat values) are plotted against the standardized residuals for each compound [54]. The Williams plots in Fig. 6, Fig. 7 show the relationship between the leverage values (expressing the similarity of a given compound to the training set) and the standardized residuals (prediction errors observed for specific compounds) for Model 1a and Model 2a, respectively. The plots are shown for average binding scores (Average BScores) and binding scores for proteins: 1BMQ, 1FM6, 1GPB, 1H5U, and 1USO.

The warning leverage (h*) is generally set to 3(*p* + 1)/*n*, where *n* is the number of training chemicals and *p* is the number of model variables (descriptors) plus one.

In the case of Model 1a, the h* value was set to 0.7, while in Model 2a, the h* value was set to 0.47.

In the Williams plot, the chemicals that are influential in the structural domain of the model are characterized by leverage (hat) value that exceeds the threshold for the warning leverage, and they should be carefully examined. The prediction errors for all compounds from the training and test sets can be illustrated with the chemicals that fall outside ± 3 standard deviation units (±3σ). Therefore, we considered chemicals outside the limits.

In Model 1a, **FD6**, **FD162,** and **FD163** are the most influential on the structural domain of the model because they are characterized by leverage (hat) value that exceeds the warning leverage threshold. **FD6** (BScores = 6922.5) has the longest alkyl chain, while **FD162** (BScores = 7417.0) and **FD163** (BScores = 7885.2) contain 6 and 8 ammonium NH4+, respectively. These chemicals are among the highly active ones. Among the chemicals outside ± 3σ, the least active unsubstituted fullerenes were found: **FD50** (BScores = 4224.3) (C70), **FD168** (BScores = 3938.3) (C60) and **FD169** (BScores = 4398.5) (C80H2).

**FD36** (BScores = 6922.5) with 2 benzene rings, 2 pyridine rings, 2 NH_2_–, 4 CH_3_–, and 4 ester groups linked to the C60 core was outside ± 3σ in the models for prediction Average BScores and binding scores for 1GPB and 1H5U.

**FD116** (BScores = 5564.4) with 6 –NO_2_ and –C = C– was found on the ± 3σ border for the model predicting binding scores for 1FM6.

**FD165** (BScores = 5975) which contains 2 phosphonate groups and 12 OH– was found outside ± 3σ for model predicting Average BScores and binding scores for 1BMQ.

**FD116** (BScores = 5564.4) with 6 –NO_2_ and –C = C– was found outside the ± 3σ limit for the model predicting the binding scores for 1FM6. **FD165** (BScores = 5975) containing 2 phosphonate groups and 12 –OH was found outside the ± 3σ limit for the model predicting Average BScores and binding scores for 1BMQ.

See Table S5 in the Supplementary Materials section for the structure of the chemicals (FDs) outside the limits for Model 1a: warning leverage threshold (h*) and outside the limit of ± 3 standard deviation units (σ).

In Model 2a, **FD4**, **FD162**, **FD163**, and **FD124** are influential on the structural domain of the model because they are characterized by a leverage (hat) value that exceeds the threshold for warning leverage. **FD4** had the long alkyl chain. **FD4** (Bscores = 7257.4) contains 8 –CH2–, 2 carboxyl –COOH, 2 amide groups. **FD162** (BScores = 7417.0) and **FD163** (BScores = 7885.2) contain 6 and 8 ammonium NH4+, respectively. **FD124** (BScores = 6650.69) consists of 2 symmetric groups with 12 –NO_2_ and 4 ketone groups.

Among the chemicals outside ± 3σ, FD6 was found to have the longest alkyl chain. This was determined for the models predicting the Average BScores, binding scores for 1BMQ, 1GPB and 1USO. **FD152** (BScores = 5909.0) was found on the border of ± 3σ in the model predicting the binding scores for 1FM6. **FD152** has 2 groups containing 4-COOH, 8–OH, 2 –C = C–. See Table S6 in the Supplementary Material*s* section for the structures of the chemicals (FDs) outside the limits for Model 2a: warning leverage threshold (h*) and outside ± 3 standard deviation units (σ).

### 3.7 Explanation of FDs interactions with proteins dependent on the structure and chemical composition of FDs and targets

A comprehensive cheminformatics analysis of structural features affecting the binding activity of fullerene derivatives is represented in our previous article [35]. The overall characteristics demonstrated that the most active FDs have the longest chain of substituents. Benzene, pyridine, and others aromatic rings also contributed to the highest binding activity, as well as the presence of cyclic groups. The lowest value of binding activity corresponds to pristine fullerene FD168 (C60). Thus, the fullerene C60 possesses the lowest values of total surface area, molecular weight, rotatable bonds, electronegative atoms, sp3 atoms, polarizability, and topological diameter [35].

In the paper [36] it was described how hydrophobicity of fullerene core along with hydrophilic interaction of side chains plays a key role in binding with the studied proteins. The authors [36] studied the contribution of a degree of hydrogen bonds, hydrophobic interactions, salt bridges, and pi interactions. The analysis of several top protein–ligand complexes revealed that a higher binding score is due to higher hydrophobic contributions from both FDs and protein, while hydrogen bond contribution from functional groups decreases the binding.

Moreover, it was reported [36] that some ligands are positioned at the outer surface of the protein. For instance, FD6 is located on the surface of the protein (blood clotting enzyme thrombin (PDB ID 1A4W)) having high binding scores. Indeed, some proteins do not have sufficient cavity space to accommodate large FDs and show the very low scores. Some FDs can be docked inside the binding pocket.

Additional docking can be done to determine the nature of interaction between selected FDs and targets in future studies.

### 3.8 Prospects for further use of obtained data

It is known that selective ligands (FDs) have a tendency to bind very limited kinds of receptors (proteins), whereas non-selective ligands bind to several types of receptors. In the paper [36] it was proposed the list of toxic FDs that are very active and bind to a large number of proteins with a high binding scores activity. The authors [36] were looking for selective FDs by visual inspection of heat map. Those FDs that had red line for majority of proteins were attributed to toxic one. (Red line in heat map corresponds to the highest values of binding scores).

In the development of drugs it is very important to take into consideration the side effects of drug candidates. In this context in the Supplementary Material 2 Excel Table we represented the heat maps. These heat maps illustrated the binding activity of all 169 FDs with 1117 proteins in the list1 and binding activity of five related to diabetic disease proteins with average binding scores of 1117 proteins in the list 2 with the indication of toxic FDs with letter T. The list of toxic FDs was taken from the study published in the article [36].

Selection of drug candidates suitable for future additional docking or/and *in vitro* study presents extensive research. Such studies need more detailed and time consuming analysis which is not in the scope of our study. But the data represented in Supplementary Material 2 Excel Table may be starting point for such research work.

The Supplementary Materials 2 (Excel Table with heat map) can be used for future search of the most promising fullerene derivatives related to anti-diabetes targets which will be useful for *in vitro* and *in vivo* investigation of FDs.

## 4 Conclusions

This article focuses on the effect of FDs on therapeutically important targets related to diabetes using chemoinformatics approaches. Prioritizing new compounds by conducting *in silico* studies limits animal testing and reduces global pharmacokinetic failures in the later stages of drug development [61].

The following results were presented in the article. A high correlation was found between binding activities related to average binding scores and binding scores for five proteins relevant to diabetes (1BMQ, 1FM6, 1GPB, 1H5U, and 1US0) ranging from 0.921 to 0.958. The correlation between the binding score activity of these five proteins (1BMQ, 1FM6, 1GPB, 1H5U, and 1US0) appeared to be in the range of 0.880–0.918.

The contribution of the most significant descriptors to protein–ligand binding activity was presented as a correlation between the descriptors and binding scores activity. Thus, the correlation between the binding activity of the five proteins associated with diabetes and the descriptors (Non-H- atoms, Rotatable Bonds, Molecular Weight, Total Surface Area, Topological Diameter, QPpolrz, and DCW) ranged from 0.657 to 0.920.

The largest contribution of protein–ligand binding (determined in the regression models) belongs to the topological diameter (TD). The size of FDs is significant for all responses: average Bscores and binding scores for five proteins associated with diabetes. This is followed by QPpolrz, which was excluded in the case of 1H5U and 1US0 proteins.

The best CPANN model 1b for prediction of binding scores activity using QPpolrz and TD descriptors is characterized by a squared regression coefficient for the training set (n = 127) R^2^ = 0.98392 (RMSE = 0.12637), for the test set (n = 42) Q^2^ = 0.99960 (RMSE = 0.01944), leave-one-out cross-validation (LOO-CV) regression coefficient, Q^2^cv = 0.97814 (RMSE = 0.14745) related to the average binding scores.

The best CPANN model 2b for prediction of binding scores activity using the optimal descriptors DCW was characterized by a squared regression coefficient for the training set (n = 127) R^2^ = 0.97968 (RMSE = 0.14205), for the test set (n = 42) Q^2^ = 0.99895 (RMSE = 0.00364), Leave-One-Out Cross-Validation (LOO-CV) regression coefficient, Q^2^cv = 0.97047, (RMSE = 0.17134) related to the average binding scores.

High correlations were also obtained for the binding scores activities of five proteins associated with diabetic diseases: R^2^ for training set ranged from 0.95 to 0.97 in Model 1b and from 0.92 to 0.96 in Model 2b; Q^2^ for test set was 0.99 in both models 1b and 2b; and LOO-CV Q^2^cv ranged from 0.93 to 0.96 in Model 1b and from 0.90 to 0.94 in Model 2b.

The models were developed in accordance with the five OECD principles.

The applicability domain was analyzed. The mechanistic interpretation contains information on the contribution of the descriptors in ligand–protein binding.

Models for prediction binding scores allow avoiding additional time-consuming calculations. The intended use of binding scores in virtual screening can be used to rank FDs to select top compounds suitable for selected disease-related proteins of interest.

It is recommended to use Supplementary Material 2 (Excel Table with heat map) for future search of the most promising fullerene derivatives related to anti-diabetes targets which will be useful for *in vitro* and *in vivo* investigation of FDs.

## Declaration of Competing Interest

The authors declare that they have no known competing financial interests or personal relationships that could have appeared to influence the work reported in this paper.
