# Supplementary material for: How fullerene derivatives (FDs) act on therapeutically important targets associated with diabetic diseases
Source: Comput Struct Biotechnol J. 2022 Feb 12;20:913–24. doi: 10.1016/j.csbj.2022.02.006 (PMC8861571; doi:10.1016/j.csbj.2022.02.006)
Supplement: Supplementary data 1 [file mmc1.docx]

How fullerene derivatives (FDs) act on therapeutically important targets associated with diabetic diseases

Natalja Fjodorova^a*^, Marjana Novic^a^, Katja Venko^a^, Viktor Drgan ^a^, Bakhtiyor Rasulev^b^, Melek Türker Saçan^c^ ; Safiye Sağ Erdem^d^; Gulcin Tugcu^e^, Alla Toropova^f^, Andrey Toropov^f^

# *Supplementary materials*

# Table of content:

**Figure S1**. The plots of actual response (binding scores) vs. predicted one for Model 1a. S3.

**Figure S2**. The plots of actual response (binding scores) vs. predicted one for Model 2a. S4.

**Figure S3.** The distribution of FDs in top map 20x20 of CPANN model overlapped with output layer with binding activity with indication of the most and least active FDs. S9.

**Figure S4**. The top map of CPANN map with indication of active FDs connected to C60 core with cyclopropane ring containing ammonium groups NH_3_^+^ and weight maps of descriptors which have the highest values in this area. S10.

**Figure S5.** The top map of CPANN map with indication of active FDs connected to C60 core with benzene ring and containing nitrogroups-NO2 and weight maps of descriptors which have the highest values in this area. S11.

**Figure S6.** The top map of CPANN map with indication of active FDs attached to the C60 core with cyclopropane 3-membered ring and containing two benzene rings and weight maps of descriptors which have the highest values in this area. S12.

**Figure S7.** The top map of CPANN map with indication of active FDs connected to C60 core with pyrrolidine 5-membered ring and containing nitroaromatic substituent and weight maps of descriptors which have the highest values in this area. S13.

**Table S1**. Statistical performance of CPANN model trained for 600 learning epochs. S2.

**Table S2**. Regression equations and performance of regression models using all descriptors including the drug like descriptors. S2.

**Table S3**. Regression equations and performance of regression models using two descriptors QPpolrz and topological descriptor (TD) related to Model 1a. S2.

**Table S4**. Regression equations and performance of regression models using Monte Carlo optimal descriptor DCW related to Model 2a. S3.

**Table S5**. Structure of chemicals (FDs) outside the limits: warning leverage threshold (h*) and outside the square area between ±3 standard deviation units (σ) in Model 1. S3-S6.

**Table S6**. Structure of chemicals (FDs) outside the limits: warning leverage threshold (h*) and outside the square area between ±3 standard deviation units (σ) in Model 2. S7-S8.

**Table S1**. Statistical performance of CP ANN model trained for 600 learning epochs.

|  | Squared Correlation coefficient R^2^ | Root Mean Squared Error RMSE |
| --- | --- | --- |
| 1. Average Binding Scores | 0.988 | 0.110 |
| 1. Binding Scores for 1BMQ | 0.973 | 0.165 |
| 1. Binding Scores for 1FM6 | 0.957 | 0.206 |
| 1. Binding Scores for 1GPB | 0.967 | 0.181 |
| 1. Binding Scores for 1H5U | 0.961 | 0.198 |
| 1. Binding Scores for 1US0 | 0.981 | 0.138 |

**Table S2**. Regression equations and performance of regression models using all descriptors including the drug like descriptors.

| Response | Regression equation | R-Sq(adj) |
| --- | --- | --- |
| **Average Bscores** | **Average Bscores** = 2464 + 17,6 DCW + 20,1 QPpolrz + 56,1 (TD) - 58,6 H-Acceptors + 3,47 Total Surface Area + 1570 Relative PSA - 8,88 Molweight + 33,3 cLogP + 60,4 Electronegative Atoms - 9,56 Stereo Centers - 79,0 Rings Closures + 35,3 Small Rings - 134 Aromatic Rings + 30,6 Aromatic Atoms + 17,1 sp3-Atoms + 95,2 Non-H Atoms | 0.968 |
| **1BMQ** | **1BMQ** = 1641 + 33,6 QPpolrz + 78,0 (TD) + 63,2 - 170 Aromatic Nitrogens - 39,1 Acidic Oxygens | 0.895 |
| **1FM6** | **1FM6** = 3953 + 27,1 QPpolrz + 98,2 (TD) + 48,4 cLogP - 242 Aromatic Rings + 43,2 Aromatic Atoms - 136 Aromatic Nitrogens | 0.857 |
| **1GPB** | **1GPB** = 2760 + 26,3 QPpolrz + 48,0 (TD) - 135 H-Acceptors + 2837 Relative PSA - 20,5 Molweight + 67,7 cLogP + 37,1 cLogS + 154 Electronegative Atoms + 36,9 Rotatable Bonds + 41,2 sp3-Atoms + 197 Non-H Atoms | 0.872 |
| **1H5U** | **1H5U** = 3435 + 35,7(TD) - 116 H-Acceptors + 115 Electronegative Atoms - 16,7 Stereo Centers + 26,4 sp3-Atoms | 0.878 |
| **1US0** | **1US0** = + 107 (TD) - 151 H-Acceptors - 102 H-Donors + 3215 Relative PSA + 5,19 Polar Surface Area - 15,9 Molweight - 20,0 Stereo Centers + 33,0 Rotatable Bonds + 43,7 Aromatic Atoms + 49,3 sp3-Atoms - 61,1 Acidic Oxygens + 263 Non-H Atoms | 0.888 |

**Table S3**. Regression equations and performance of regression models using two descriptors QPpolrz and topological descriptor (TD) related to Model 1a.

| Response | Regression equation | R-Sq(adj) |
| --- | --- | --- |
| **Average Bscores** | Average Bscores = 1516 + 42,5 QPpolrz + 73,0 (TD) | 0.93 |
| **1BMQ** | 1BMQ = 1101 + 35,6 QPpolrz + 83,8 (TD) | 0.87 |
| **1FM6** | 1FM6 = 3731 + 24,4 QPpolrz + 98,6 (TD) | 0.83 |
| **1GPB** | 1GPB = 2920 + 30,0 QPpolrz + 72,9 (TD) | 0.81 |
| **1H5U** | 1H5U = 2143 + 40,8 QPpolrz + 64,5 (TD) | 0.84 |
| **1US0** | 1US0 = 687 + 48,0 QPpolrz + 75,7 (TD) | 0.84 |

**Table S4**. Regression equations and performance of regression models using Monte Carlo optimal descriptor DCW related to Model 2a.

| Response | Regression equation | R-Sq(adj) |
| --- | --- | --- |
| **Average Bscores** | Average Bscores = 309 + 76,8 DCW | 0.93 |
| **1BMQ** | 1BMQ = - 80 + 71,8 DCW | 0.85 |
| **1FM6** | 1FM6 = 2701 + 61,4 DCW | 0.74 |
| **1GPB** | 1GPB = 1927 + 61,0 DCW | 0.77 |
| **1H5U** | 1H5U = 1008 + 72,2 DCW | 0.84 |
| **1US0** | 1US0 = - 565 + 83,6 DCW | 0.82 |


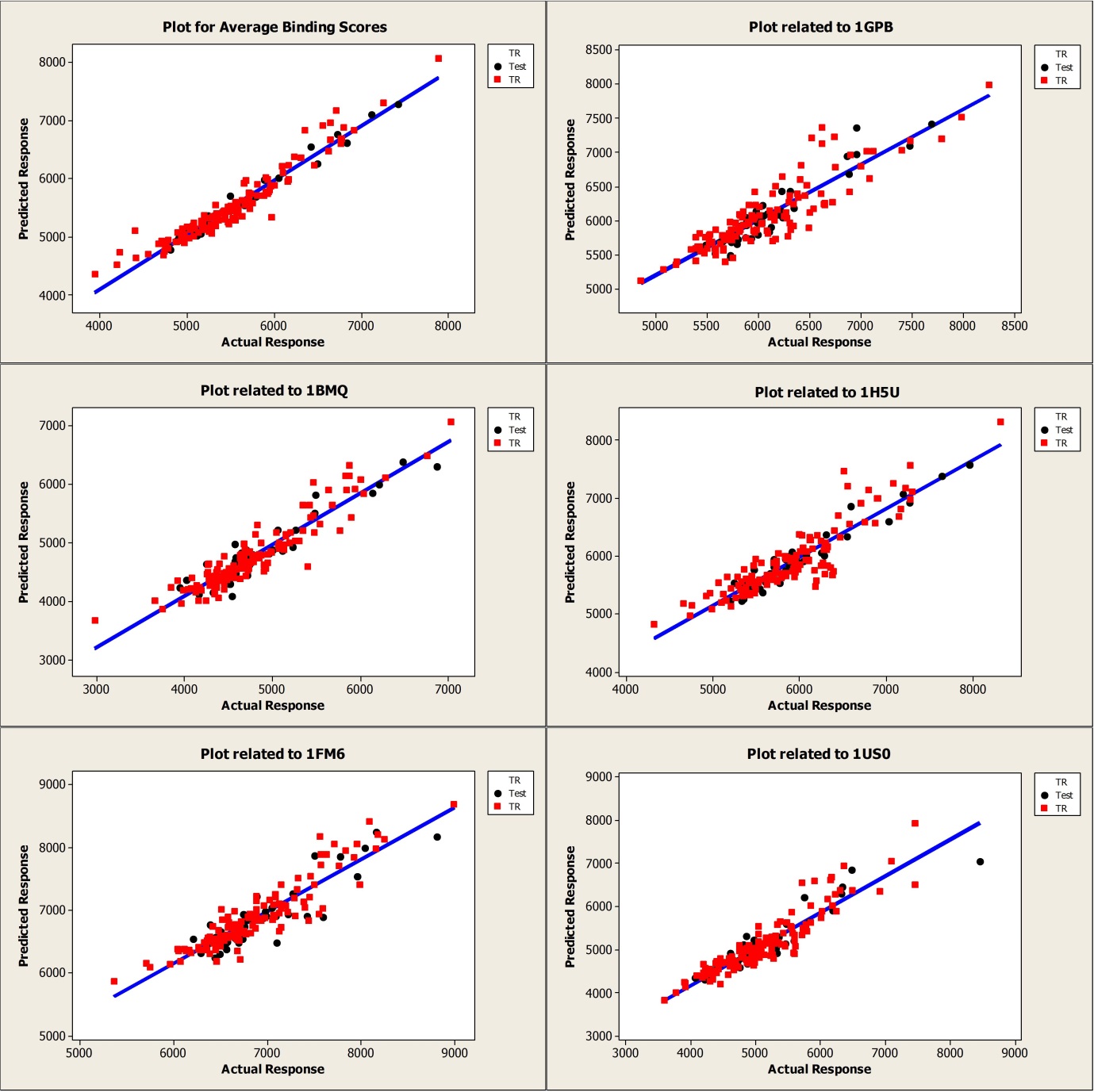


**Figure S1**. The plots of actual response (binding scores) vs. predicted one for Model1a.


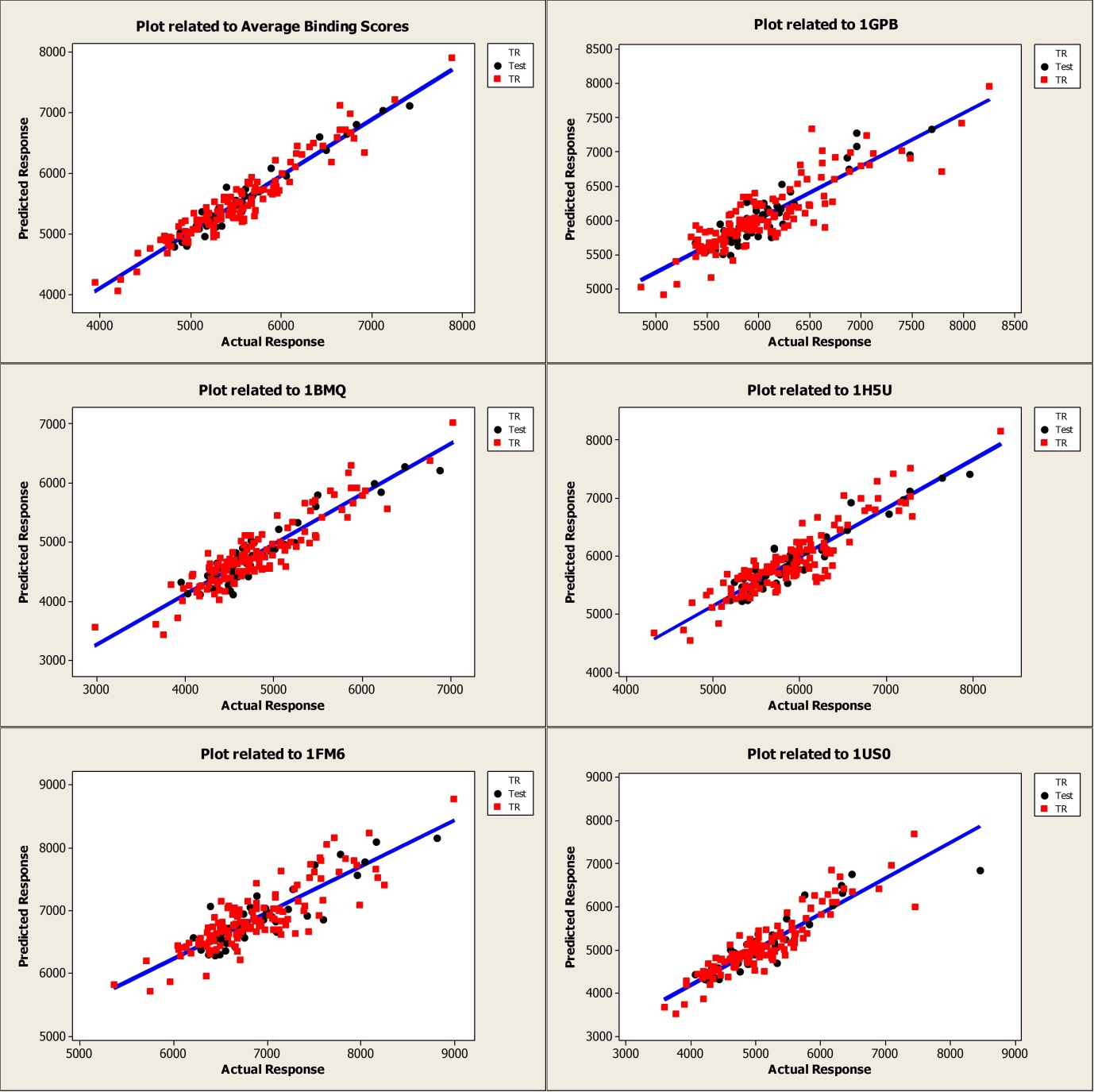


**Figure S2**. The plots of actual response (binding scores) vs. predicted one for Model 2a.

**Table S5**. Structure of chemicals (FDs) outside the limits: warning leverage threshold (h*) and outside the square area between ±3 standard deviation units (σ) in Model 1a.

| FDs outside h* | FDs outside ±3 σ |
| --- | --- |
|   **FD6** (BScores=6922.5) has the longest alkyl chain with alkenyl group -C=C- and 1 -COOH, -NH- | 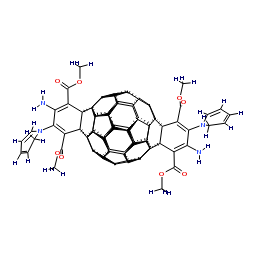  **FD36** (BScores=6922.5) connected to C60 core with 2 benzene rings and containing 2 pyridine rings, 2-NH2, 4-CH3, 4 esther groups;  2groups below   |
| 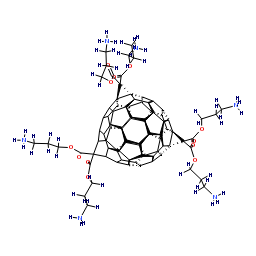  **FD162** (BScores=7417,0) consists from 3 groups below with 6NH3  | 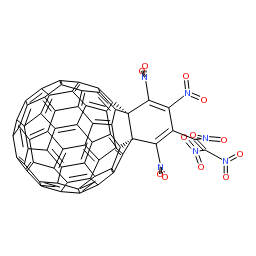  **FD116** (BScores=5564.4) consists of 6-NO2, -C=C-   |
| 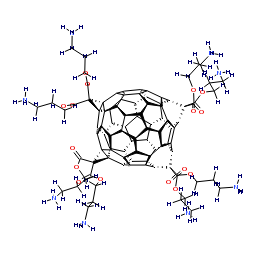  **FD163** (BScores=7885,2) consists of 4 groups below with 8NH3 | 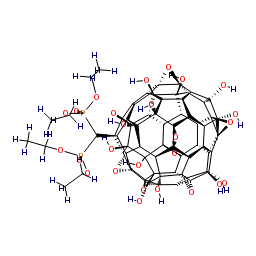  **FD165-**(BScores=5975) contains 2phosphonate groups and 12 -OH   |
|  | 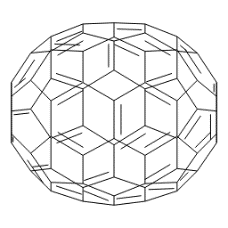  **FD50** (BScores=4224,3)**-**pristine fullerene C70  Least active without functional groups |
|  | 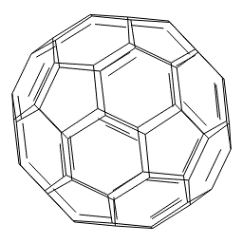  **FD168**-(BScores=3938,3)-pristine fullerene C60. Least active without functional groups |
|  |   **FD169** (BScores=4398.5) fullerene C80H2  Least active without functional groups |

**Table S6**. Structure of chemicals (FDs) outside the limits: warning leverage threshold (h*) and outside the square area between ±3 standard deviation units (σ) in Model 2a.

| FDs outside h* | FDs outside ±3 σ |
| --- | --- |
|   **FD4-** (Bscores= 7257.4) containe 8-CH2-; 2carboxyl-COOH;  2amide groups; |   **FD6** (BScores=6922.5) has the longest alkyl chain with alkenyl group -C=C- and 1 -COOH, -NH- |
| 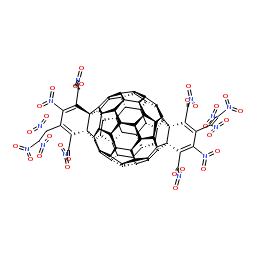  **FD124**-(BScores=6650.69- 2 groups below: 12-NO2; 4 ketone groups   | 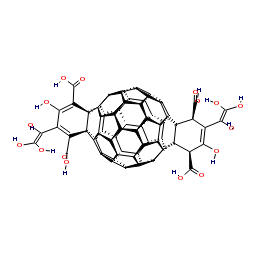  **FD152**-(BScores=5909,0)- 2 groups below :4-COOH, 8-OH, 2-C=C-   |
| 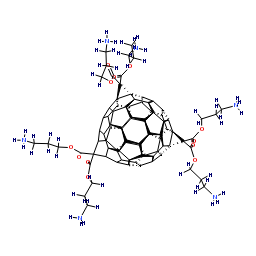  **FD162** (BScores=7417,0) consists from 3 groups below with 6NH3   |  |
| 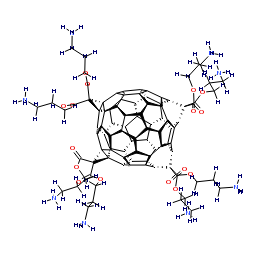  **FD163** (BScores=7885,2) consists of 4 groups below with 8NH3   |  |


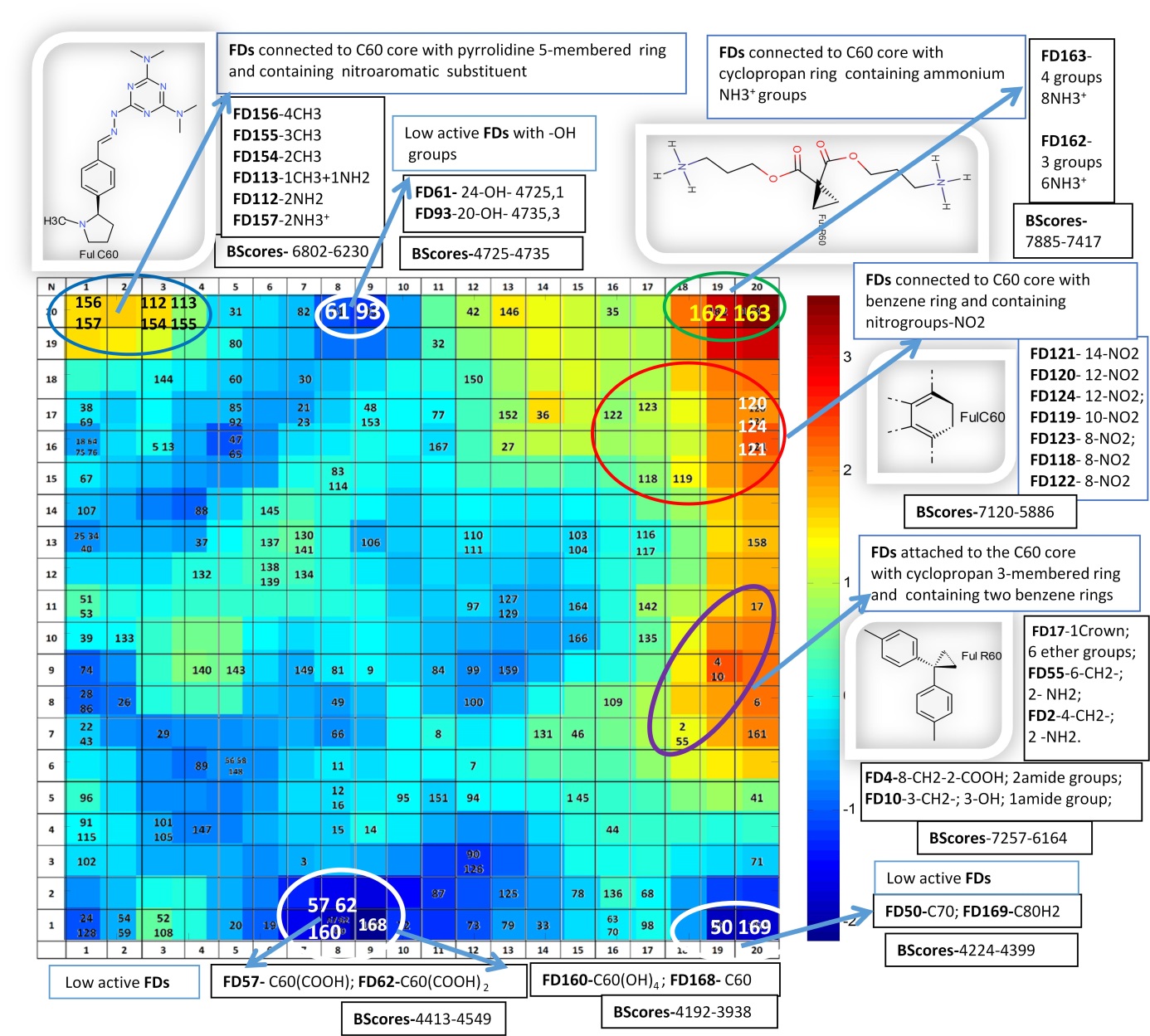


**Figure S3.** The distribution of FDs in the top map 20x20 of CPANN model overlapped with output layer with binding activity with indication of the most and least active FDs.

The distribution of FDs in top map 20x20 of CPANN model overlapped with output layer with binding activity with indication of the most and least active FDs is shown in Figure S3.


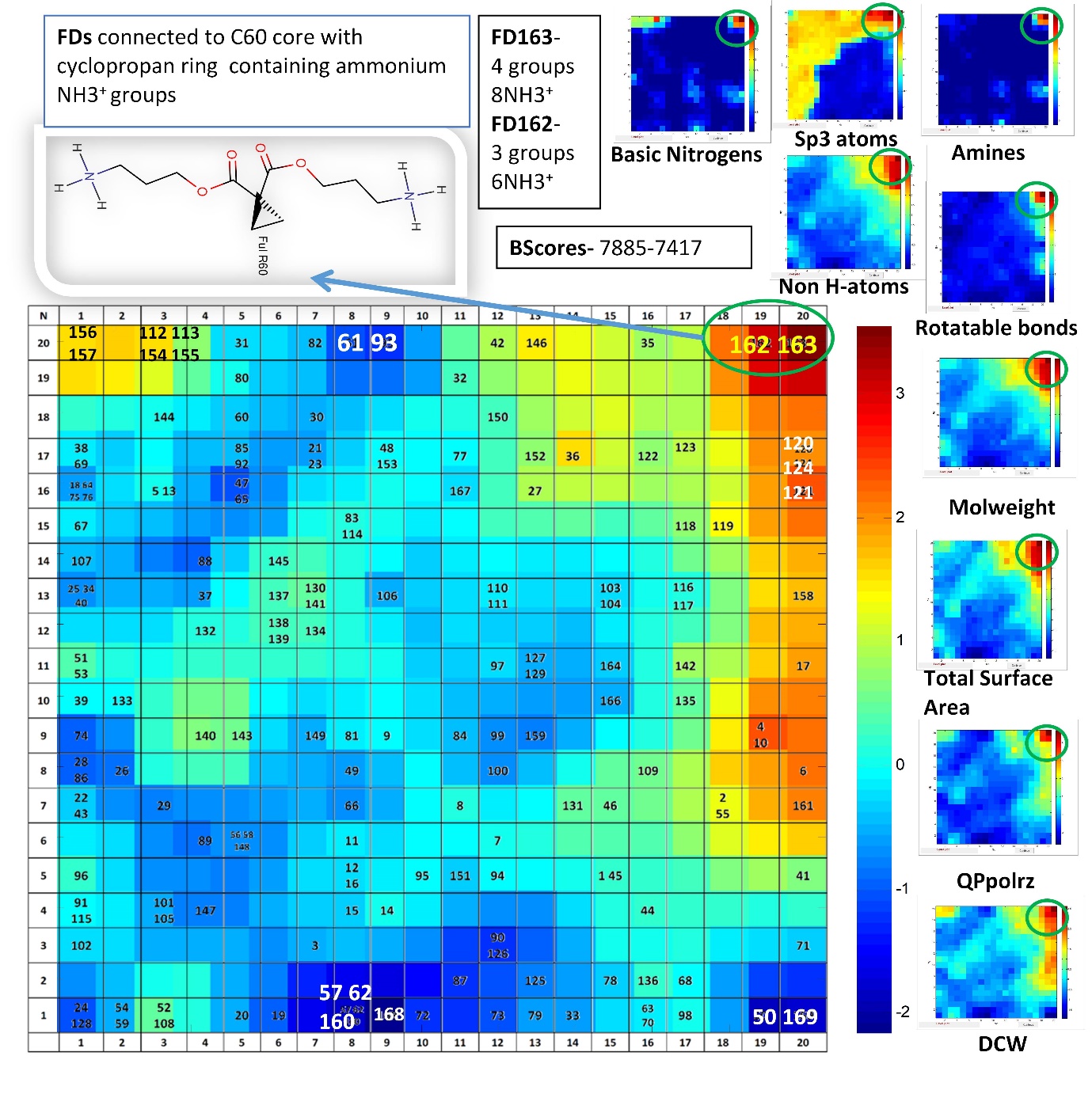


**Figure S4**. The top map of CPANN map with indication of active FDs (GROUP A) connected to C60 core with cyclopropane ring containing ammonium groups NH_3_^+^ and weight maps of descriptors which have the highest values in this area.

The active FDs (GROUP A) connected to C60 core with cyclopropane ring containing ammonium groups NH_3_^+^ which were located in neurons 19x20 and 20x20 marked with green circle have the highest values for descriptors: Basic Nitrogens, Sp3 atoms, Amines, Non H-atoms, Rotatable bonds, Molweight, Total Surface Area, QPpolrz, DCW.

**
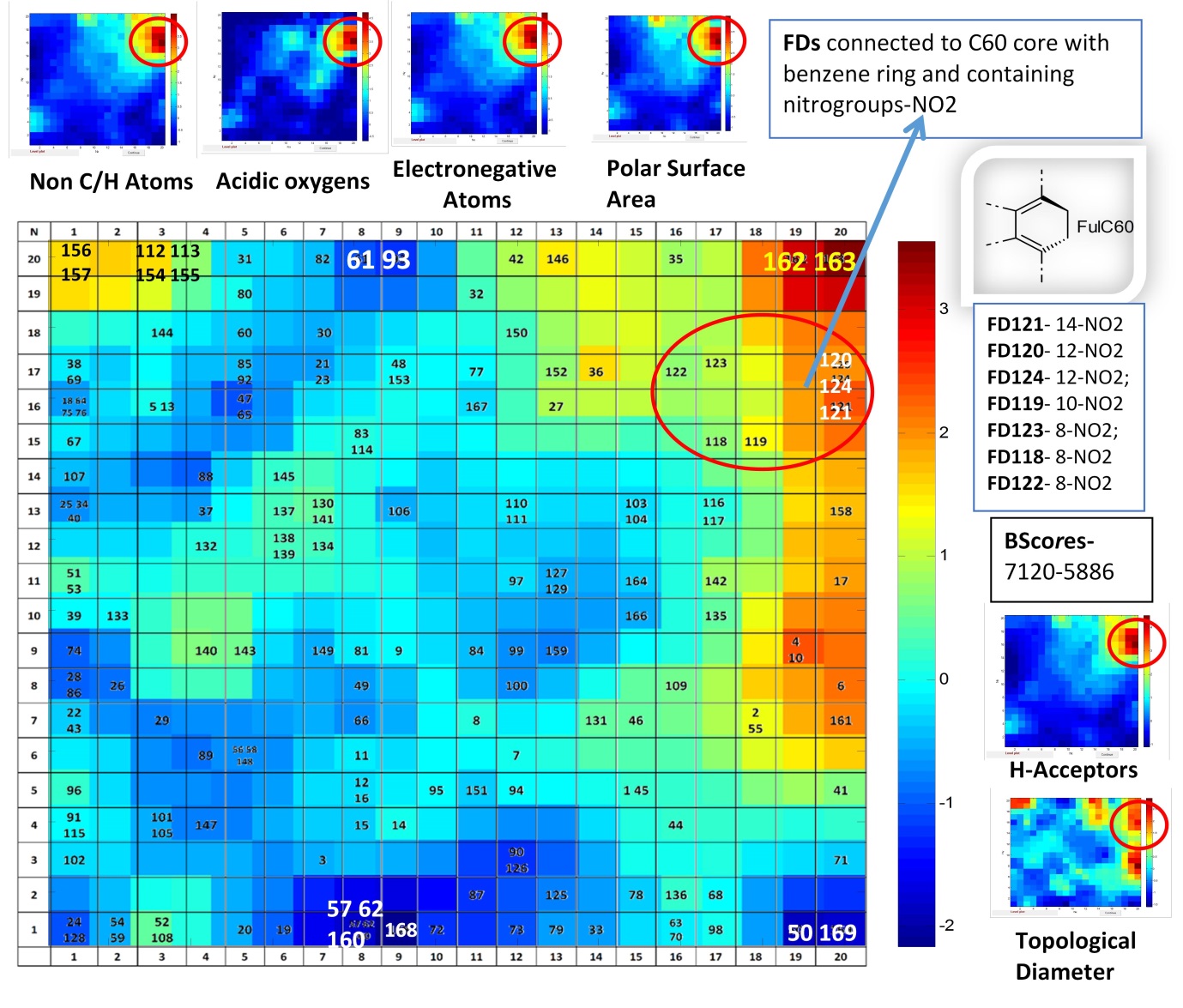
**

**Figure S5.** The top map of CPANN map with indication of active FDs (GROUP B) connected to C60 core with benzene ring and containing nitrogroups-NO2 and weight maps of descriptors which have the highest values in this area.

The active FDs (GROUP B) connected to C60 core with benzene ring and containing nitrogroups-NO2 which were located in neurons marked with red circle have the highest values for descriptors: Non C/H Atoms, Acidic oxygens, Electronegative Atoms, Polar Surface Area, H-Acceptors, and Topological Diameter.

**
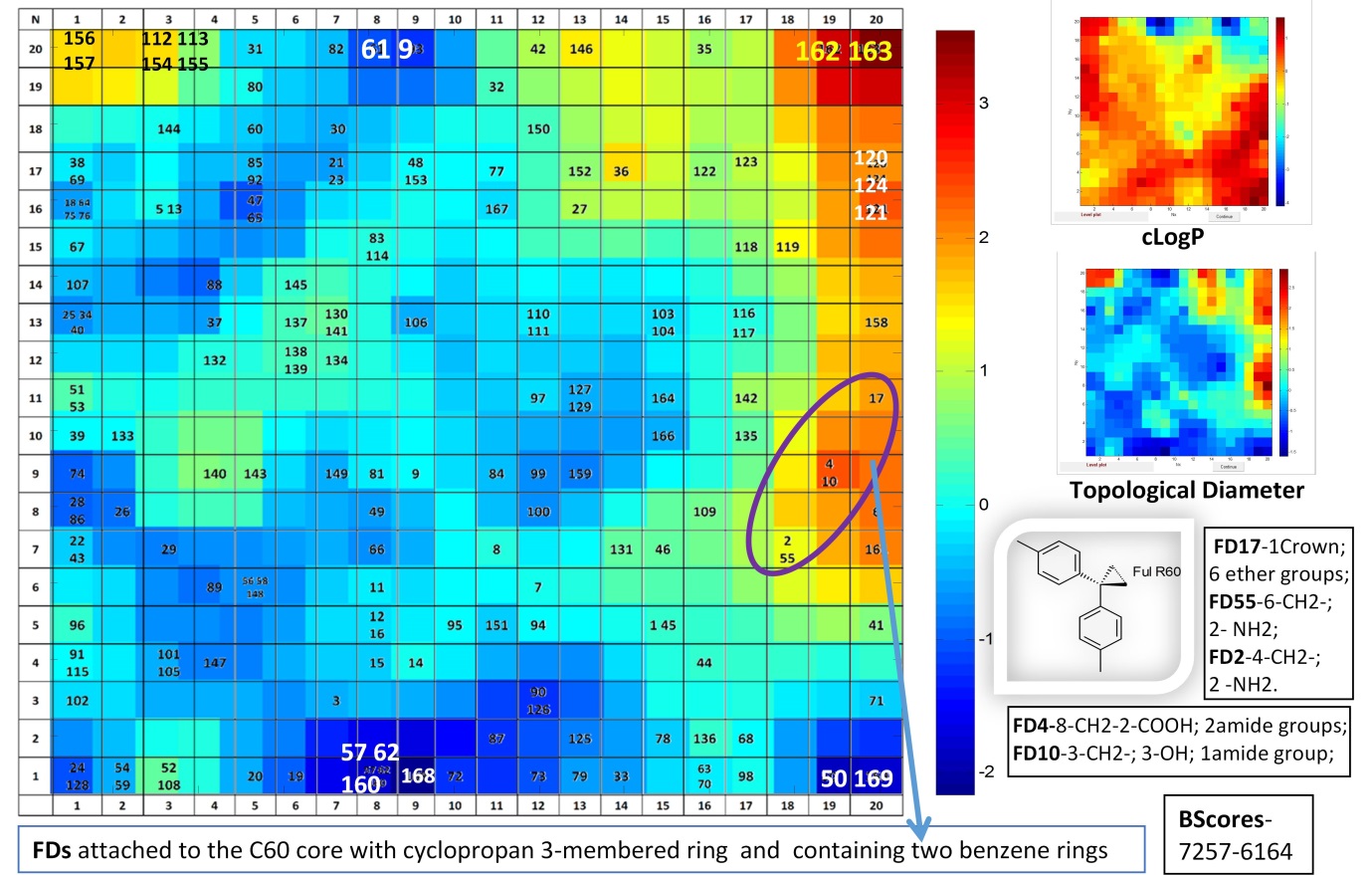
**

**Figure S6.** The top map of CPANN map with indication of active FDs (GROUP C) attached to the C60 core with cyclopropane 3-membered ring and containing two benzene rings and weight maps of descriptors which have the highest values in this area.

The active FDs (GROUP C) attached to the C60 core with cyclopropane 3-membered ring and containing two benzene rings which were located in neurons marked with blue circle have the highest values for descriptors: cLogP, Topological Diameter.

These two benzene rings are related to endocrine disruptor structural alert.


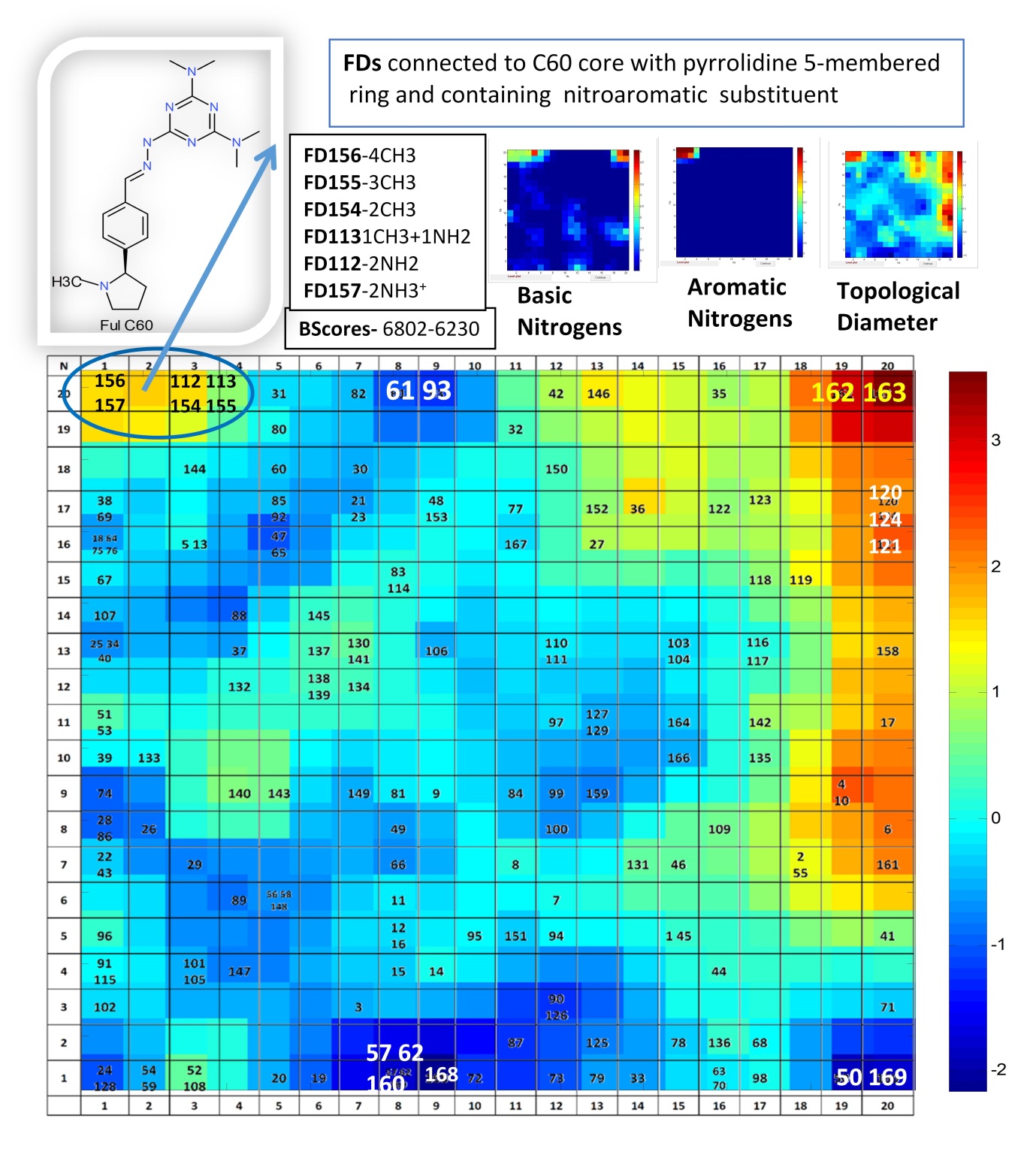


**Figure S7.** The top map of CPANN map with indication of active FDs connected to C60 core with pyrrolidine 5-membered ring and containing nitroaromatic substituent and weight maps of descriptors which have the highest values in this area.

The active FDs connected to C60 core with pyrrolidine 5-membered ring and containing nitroaromatic substituent which were located in neurons marked with blue circle have the highest values for descriptors: Basic Nitrogens, Aromatic Nitrogens, Topological Diameter.
